# Computational Fluid Dynamics data for improving freeze-dryers design

**DOI:** 10.1016/j.dib.2018.05.141

**Published:** 2018-05-31

**Authors:** Antonello A. Barresi, Daniele L. Marchisio

**Affiliations:** Politecnico di Torino, Department of Applied Science and Technology, C.so Duca degli Abruzzi 24, I-10129 Torino, Italy

## Abstract

Computational Fluid Dynamics (CFD) can be used to simulate different parts of an industrial freeze-drying equipment and to properly design them; in particular data concerning the freeze-dryer chamber and the duct connecting the chamber with the condenser, with the valves and vanes eventually present are given here, and can be used to understand the behavior of the apparatus allowing an improved design. Pilot and large scale freeze-drying chambers have been considered; data of a detailed simulation of a complete pilot scale apparatus, including duct and condenser, are included. Data on conductance of an empty duct with different L/D ratio, on disk valves with different geometry, and on mushroom valve are presented. Velocity, pressure, temperature and composition fields are reported on selected planes for chambers and valves. Results of dynamic simulations are also presented, to evaluate possible performance of monitoring devices in the chamber. Some further data, with detailed interpretation and discussion of the presented data can be found in the related research article by Barresi et al. [1] and Marchisio et al. [2].

**Specifications Table**

**Table:** 

Subject area	Chemical engineering
More specific subject area	Fluid dynamics, Freeze-drying, Pharmaceutical technology
Type of data	Table, image (CFD contour plot), text file, graph, figure
How data was acquired	CFD simulations
Data format	Analyzed
Experimental factors	Velocity, pressure, temperature and composition fields reported on selected planes for chamber and valves; correlations for pressure drop in the chamber; response analysis of virtual sensors; correlations for duct and valve mass flow conductance
Experimental features	Pilot and large scale freeze-drying chamber; detailed simulation of complete pilot scale apparatus, including duct and condenser; empty ducts with different L/D ratio, disk valves with different geometry, mushroom valve
Data source location	N/A
Data accessibility	Data available with article

**Value of the data**

• Data show how Computational Fluid Dynamics can be used as support to the design of a freeze-dryer and scale-up of cycles.
• Pressure and gas composition variations in the chamber, which can affect the batch uniformity, are quantitatively evaluated.
• Pressure drop and maximum allowable flow in duct and through butterfly valves are given in form of correlations suitable for design.
• Correlations for pressure drop and maximum flow for mushroom valves, as a function of the valve opening distance are given.
• Data for critical mass flow are shown in term of mass flux, to allow validation with experimental data.

## 1 Data

Data concerning the influence of chamber geometry, number of shelves and clearance between shelves, position of the duct leading to the condenser, number and position of the inert gas injection nozzles on fluid dynamics, water mass fraction and pressure distribution in the chamber, are first presented. Data on fluid dynamics of ducts and valves, and of a pilot scale complete apparatus, are then shown. The effect of the boundary conditions selected (including the slip or no-slip assumption), depending on the Knudsen number, and of actual entrance conditions is also documented. Graphs for estimation of critical mass flow in empty ducts and in presence of butterfly and mushroom valves are given. Design charts and correlations for equivalent length of valves developed from data here presented are given and discussed in Ref. [2]. Table 1 summarizes the simulation conditions adopted, the variables considered and the data made available.

**Table 1:** Synopsis of simulation conditions adopted, variables considered and data made available.

**Apparatus**	**Type of simulation**				**Variables investigated**	**Data available**
	**SS no-slip b.c.**	**SS slip b.c.**	**wall thermal b.c.**	**transient (no slip)**		
Pilot scale freeze-dryer chamber	x	x	x	x	– duct position – inter-shelves clearance – sublimation rate – inert gas flow rate	Fluid dynamics
						Pressure distribution
						Gas temperature field
						Response to disturbance by virtual sensors in different locations
						Inert gas distribution
						Influence on product drying
Large scale freeze-dryer chamber and duct	x				– number ofshelves (shelves clearance) – sublimation rate – chamber ref pressure	Fluid dynamics
						Pressure distribution
						Pressure drop in chamber and duct
						Velocity profiles in the duct
Pilot scale apparatus (chamber, duct & valve, condenser)		x			– rounded andsharp duct entrance – sublimation rate	Velocity & pressure profiles in duct and condenser
						Water mass fraction distribution in condenser
Straight duct	x	x	x		– L/D ratio – inert gas fraction	Design chart
						Critical mass flux
						Comparison with “jet flow” calculations
Butterfly valve	x	x	x		– valve shape (flat & rounded)	Fluid dynamics
						Critical mass flux
Mushroom valve		x			– valve disk distance – sublimation rate	Pressure and velocity field
						Critical mass flux

SS: steady state simulation; b.c.: boundary condition.

## 2 Experimental design, materials and methods

### 2.1 Case studies

#### 2.1.1 Freeze-dryer chamber

**Fig. 1:**
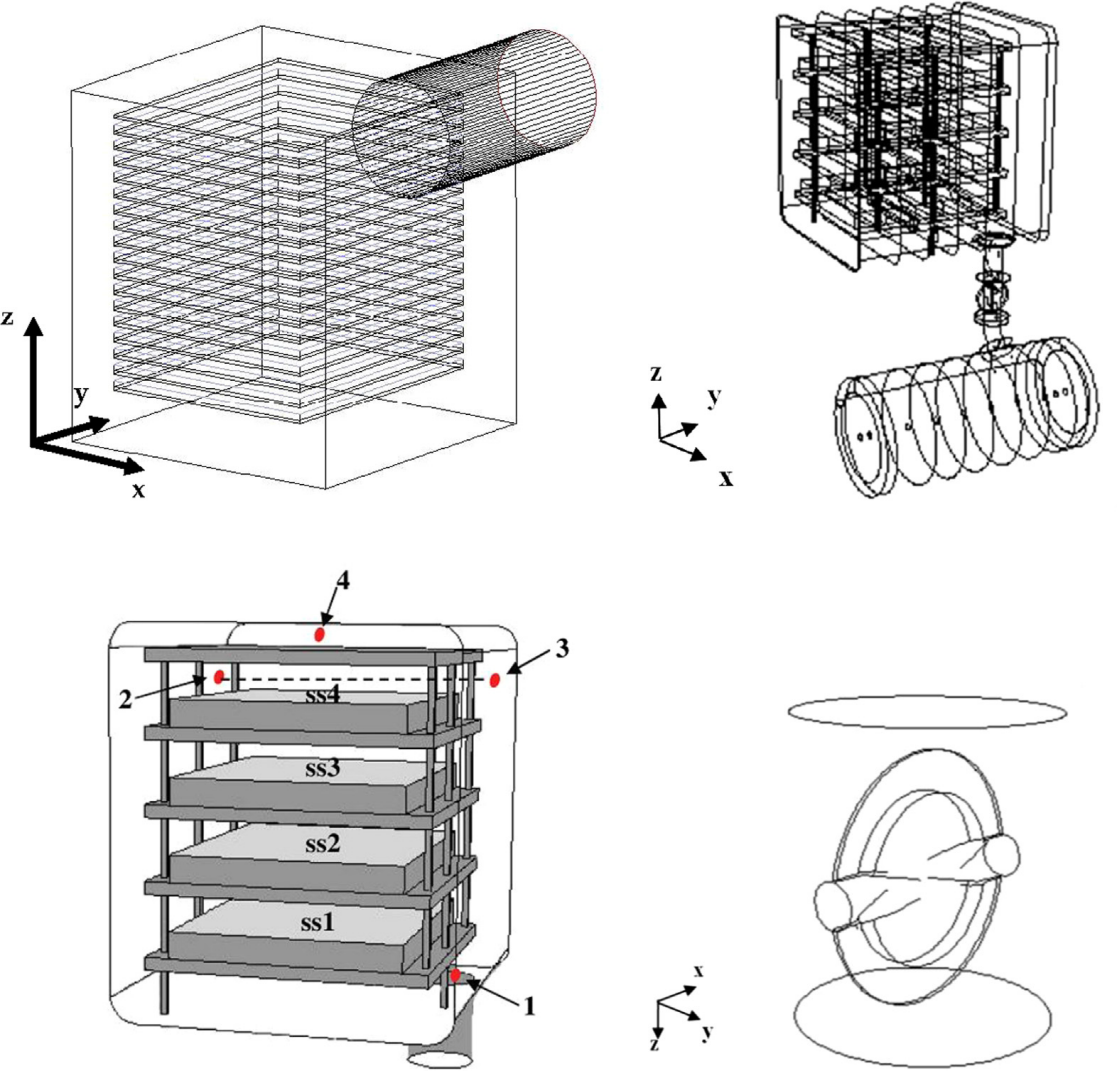
In the upper row, the two freeze-dryer chambers modelled. Left graph: the large-scale freeze-dryer, simplified from the actual *LyoMega*^TM^ 400 by Telstar; the configuration with 17 active shelves (1500×1800 mm^2^ each) and a top shelf is shown, but cases with lower shelf number have been also considered. Right graph: the pilot scale, corresponding to the *LyoBeta*^TM^ 25 by Telstar, with four shelves (450×455 mm^2^ each), a curved duct with butterfly valve, and a cylindrical condenser; cases with four shelves but different clearances between them have been also considered. In the bottom left side, the detail of the pilot scale chamber, with the location of the four different ideal sensor positions considered in the scalar transient simulations is shown (the dynamic response to a step disturb in the sublimation flux, *ss*_*i*_, is investigated). In the bottom right side the detail of the butterfly valve with profiled disk.

Two different equipment sizes (a small-scale apparatus and an industrial-scale apparatus) have been investigated (see Fig. 1). For the small-scale apparatus, three different positions for the duct have been considered: in the center of the rear wall, on one side of the rear wall and on the bottom of the chamber (details of the geometries are shown together with the fluid dynamic data). The laboratory-scale freeze-dryer is constituted by four shelves (450×455 mm^2^) for a total chamber volume of 0.2 m^3^. The large-scale freeze-dryer contained 14–17 shelves of 1500×1800 mm^2^ for a total volume of 10.3 m^3^ (with a 200 mm lateral channel) and a 800 mm diameter horizontal duct (duct length/duct diameter=2).

Three different distances between the product and the upper shelf have been considered for the small-scale apparatus (with 4 shelves) and four for the large-scale apparatus. In the industrial chamber the number of usable shelves has been varied from 14 to 17, but the position of the first and of the last shelf was not varied. In Table 2 the values of the distance between the shelves, *h*, and of the clearance between the product and the upper shelf, *r*, considered for the simulations for both the small- and large-scale apparatus have been reported.

**Table 2:** Geometrical characteristics of the (L) industrial and (S) pilot scale drying chamber configurations.

**Case**	**Number of shelves**	**Shelf-shelf distance,*****h*****(mm)**	**Product-shelf distance,*****r*****(mm)**
L1	14 (+1)	110	67
L2	15 (+1)	100	57
L3	16 (+1)	93.5	50.5
L4	17 (+1)	85	42
S1	4 (+1)	100	57
S2	4 (+1)	60	17
S3	4 (+1)	50	7

#### 2.1.2 Duct and valves

Different length to diameter ratios (*L*/*D*) have been considered for the empty duct, from 1.2 to 50, with DN=700 mm.

As concerns the butterfly valve simulations, just a short piece of straight duct 830 mm long and with a diameter of 700 mm has been considered, with a butterfly valve in open position. Two different valve shapes have been analyzed: in a first series of simulations a simplified geometry has been considered, represented by a round disk (685 mm in diameter) with uniform thickness assumed equal to 40 mm. Then the butterfly valve (again represented in open position) has been reproduced more faithfully to its real geometry (see Fig. 1). The overall diameter of the valve is still 685 mm, but the valve is represented as the union of three rings of different diameter: an outer ring about 80 mm wide (from radius 342.5 mm to radius 264.4 mm), presenting a thickness of 12 mm at the valve outer border, that increases linearly up to 16 mm; an intermediate ring with a starting thickness of 16 mm (at the radius of 264.4 mm) and a final thickness of 52 mm (at the radius 235 mm), with a non-linear profile; and a central “spherical cap” with a radius of 235 mm, with a starting thickness of 52 mm (at radius 235 mm) and reaching a maximum thickness of 125 mm in its center. These thickness values refer to the whole valve but it is also necessary to consider the intersection of the valve with the pipe used for its movement, presenting a diameter of 125 mm.

The mushroom valve is modelled as a flat disk (diameter=750 mm; thickness=80 mm). A standard horizontal condenser, characterized by a standard toro-spherical bottom (DIN 28011) and a conical inlet, equipped with a mushroom valve, has been considered. The smallest section is at the intersection of the entrance cone with the vessel bottom, and has a diameter *D*=700 mm; this geometry is compatible with the industrial freeze dryer considered. Only the first part of the apparatus has been considered, where there is no ice formation, and thus no material sink.

#### 2.1.3 Complete pilot-scale freeze-dryer

Some simulations have been carried out on the whole pilot-scale apparatus (*LyoBeta*^™^ from Telstar, Terrassa, Spain), including the real chamber described previously, the duct and also the condenser (see Fig. 1). A simple flat disk butterfly valve has been considered in the duct, which ends with a bend in the cylindrical condenser. Two different chamber-duct connections have been compared, a sharp entrance and a rounded one, corresponding to the actual realization (details of the meshes will be shown with the data).

### 2.2 Computational details

Governing equations used in the CFD model can be found in the research articles [1], [2].

The three-dimensional simulations carried out to obtain the data presented are based on structured computational grids of hexahedral cells, representing the geometry of the freeze-drying chamber, by using standard numerical methods; successive grid refinement was used to verify the grid independence of the solution for all the cases investigated. All these pieces of equipment, geometrically reproduced and meshed, have been modelled by means of Computational Fluid Dynamics, with the commercial CFD code Ansys Fluent that, in certain cases, has been coupled to User Defined Functions (UDF) developed on purpose.

The continuity and Navier–Stokes equations are solved by resorting to a finite-volume discretization. The Semi-Implicit Method for Pressure-Linked Equations (SIMPLE) algorithm was used to solve the pressure-velocity coupling, whereas, in order to contrast the insidious effects of numerical diffusion, the Quadratic Upwind Interpolation for Convective Kinematics (QUICK) was used, by refining an initial solution obtained with a first-order upwind interpolating scheme [3]. Very restrictive convergence criteria were used, with normalized residuals smaller than 10^−6^, and, in some cases, small under-relaxation factors needed to be used to reach convergence.

It is important to highlight here that for the operating conditions considered, the Knudsen number, evaluated estimating the vapor mean free path from kinetic theory and using the shelf clearance as characteristic dimension, was generally verified to be sufficiently small (of the order of 0.01) to assume that the gas flows in the continuum regime. For a number of cases (in particular for the small scale apparatus with the smallest clearance, where the *Kn* was higher) and for the butterfly valves, the possibility of imposing slip boundary conditions on the walls (typical of the transition regime between the continuum and molecular flow) was tested. As the results evidenced that, for the geometries and the operating conditions investigated, this has little effect on the predictions of the pressure drops, and almost no effect on the flow field predictions, the main part of the simulations and in particular those for the large scale apparatus were carried out with no-slip boundary conditions.

Steady-state simulations were generally carried out, considering the water vapor as a compressible fluid, whose density is evaluated according to the ideal gas law, whereas the viscosity, *μ*, is calculated with the standard kinetic theory [4]:

(1) $$\mu \;=\frac{2}{3}\frac{\sqrt{Mk_{B}T}}{\sqrt{\pi }d^{2}}$$

where *M* is the mass of the molecule (expressed in kg), *k*_*B*_ is the Boltzmann constant (1.38066·10^−23^ J K^−1^), *T* is the temperature (K) and *d* is the hard-sphere parameter for the molecule of mass *M*.

Flow field data have been also reported in dimensionless form, as Mach number (*Ma*), defined as the ratio between the gas velocity and the sound speed estimated as follows:

(2) $$Ma=\frac{U}{a}=\frac{U}{\sqrt{kRT}}$$

where *U* is the flow velocity and *a* is the speed of sound of the fluid (*a*=$\sqrt{kRT}$). From the ideal gas theory a value *k*=*c*_*p*_/*c*_*v*_=4/3 is assumed for the specific heat ratio of water vapor.

#### 2.2.1 Freeze-dryer chamber

The grid was made of about 300,000 (for the small-scale apparatus chamber) or 600,000 (for the large-scale apparatus) cells. Inlet boundary conditions were set for the sublimation surfaces, corresponding to the vials placed on each shelf, while a standard pressure-outlet boundary condition was used for the final section of the duct connecting the chamber to the condenser.

The layer of vials has been modelled as a slab, with a thickness of 43 mm corresponding to that of the vials partially stoppered, with an equivalent uniform vapor source on the upper side.

Preliminary simulations showed that the flow field predicted with detailed (array of vials) and simplified (slab) mass-flow-inlets is identical, mainly due to the pressure drop that the water vapor has to overcome to flow through the clearance between the shelves [5]. Therefore, the simple continuous slabs have been considered as mass-flow-inlet boundary conditions. A uniform mass flux was thus considered at the upper surface of the slab, corresponding to the actual average sublimation flux obtained over the shelf (that depends on the real sublimation rate per unit surface of product, on the vial arrangement, and vial wall thickness).

Most of the cases investigated included only water vapor as solvent, but in a few selected test cases injection of inert gases (i.e. nitrogen) at different flow rates to control the chamber pressure, was also simulated.

The CFD simulations have been run considering typical values of the operating parameters: operating pressure of 10 Pa (value imposed at the end of the portion of the duct considered in these simulations) has been generally set, but a few simulations have been carried out also at different pressures.

The chamber walls were considered at 283 K, with a shelf temperature of 258 K. In some simulations concerning the whole apparatus the case of adiabatic walls has been also considered; in any case, it must be noted that the condition at the external walls does not influence the pressure distribution over the shelves, which is strongly dependent on the clearance, pressure, and sublimation rate.

Steady-state simulations of the primary-drying phase have been carried out considering the maximum available sublimation rate of 1 kg h^−1^ m^−2^ with an interface product temperature of 239 K; other simulations have been also carried out at different sublimation rates (from 0.50 kg h^−1^ m^−2^ to 0.90 kg h^−1^ m^−2^). The temperature of the vapor was assumed equal to the temperature of the sublimating surface (*T*_*i*_).

For the simulations concerning the mushroom valve section, the data refer to the maximum flow estimated for the large scale equipment: in particular the configuration with the maximum number of shelves (17), corresponding to 54 m^2^, and the conventional maximum sublimation rate (1 kg h^−1^ m^2^), leading to 0.015 kg s^−1^ has been considered as reference.

Some scalar transient simulations have been also carried out to investigate the dynamic response of the small apparatus to variations of the sublimation rate on the different shelves.

As concerns the effect of radiation on the gas fluid dynamics, radiation contribution of the gas has been also taken into account by means of P1 radiation model. The P1 radiation model is the simplest case of the P-N model, which solves the radiation transfer equation (RTE) by means of the expansion of the radiation intensity into an orthogonal series of spherical harmonics.

#### 2.2.2 Choked flow in ducts

For the duct simulations (either empty or with the butterfly valve), the inlet of the pipe is modelled as a pressure inlet (the stagnation pressure and temperature are imposed and calculated by assuming an isentropic compression), whereas the outlet section is modelled as a pressure outlet (the outlet pressure is imposed), considering for all the cases an inlet static temperature of 239 K.

Simulations are three-dimensional in space and steady-state in time. A preliminary study was carried out to establish the grid size for which no influence on the results was observed; a mesh with 80,000 nodes was necessary for the longest duct (*L*/*D*=50). The convergence criteria for the residuals were set at 10^−6^.

In order to determine the critical mass flow, a series of simulations has been carried out at constant inlet pressure and different values for the outlet pressure, reducing progressively the outlet pressure, to identify the value at which no further increase in the flow occurred; the results confirmed that the critical flow conditions could be reliably estimated. Then different inlet pressures have been investigated, repeating the previous procedure. It must be evidenced that certain static pressures, and Mach numbers, have been supposed on the inlet and the relative stagnation conditions have been calculated, but inlet and outlet pressure given at the beginning are considered approximated values, and are adjusted by the code: a relevant number of simulations have been lunched to get a good estimation of the critical conditions.

**Fig. 2:**
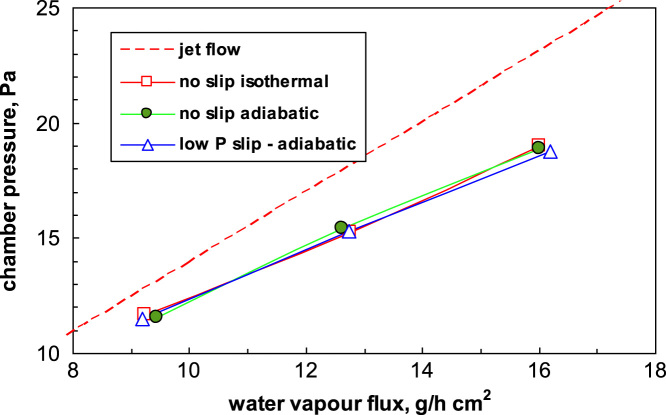
Influence of boundary conditions on estimated critical flow. Straight duct, *L/D*=2.

To investigate the relevance of the boundary conditions (adiabatic, or isothermal wall at 239 K) some simulations have been carried out; a small influence of the boundary conditions was observed (see Fig. 2); it has to be noted that for the wall the same temperature of the gas was used, but the inclusion of heat supply from the wall would make the results very dependent on the case considered. The jet flow calculations in this case underestimate the conductance of the duct, but only because null inlet velocity is considered. Due to the low pressure and short residence time, the assumption of adiabatic flow is realistic. Low pressure slip boundary conditions, that should be applied in case of transitional regime (as discussed in Ref. [1], have been also tested, but the results confirm that the assumption of viscous flow (and thus the no-slip boundary conditions)) are acceptable at least for larger ducts.

**Fig. 3:**
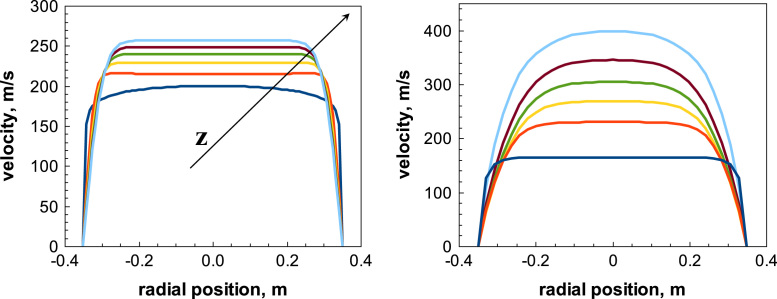
Development of radial velocity profiles in a duct (DN 700). Left graph: *L*/*D*=2, subsonic; right graph: *L*/*D*=50, sonic. Dark blue line corresponds to inlet, light blue to outlet: intermediate profiles at regular distances are also shown.

An almost flat velocity profile is considered at the inlet of the duct, as a uniform pressure is imposed. The velocity profile will develop in the duct, even if for small *L/D* values the profile at the exit will be still far from the parabolic one corresponding to fully developed flow. An example is shown in Fig. 3. In the short duct the profile remains flat, while in the long duct the velocity can increase, reaching sonic conditions, and a parabolic profile can develop.

In all cases the absolute (static) pressure is given, but it must be considered that two different values can be considered:

– the "contour pressure", that is the value measured on the contour of the duct. For the inlet it will give the maximum value at the entrance, and for the outlet the minimum value at the exit.
– the "surface average pressure", that is the average value calculated on the whole surface considered. The data presented will generally refer to this pressure.

#### 2.2.3 Conductance of butterfly valves

A procedure similar to that previously described for the empty duct was used for the simulation of the piece of duct containing the butterfly valve. The computational mesh used in the CFD simulations contains about 650,000 cells.

**Fig. 4:**
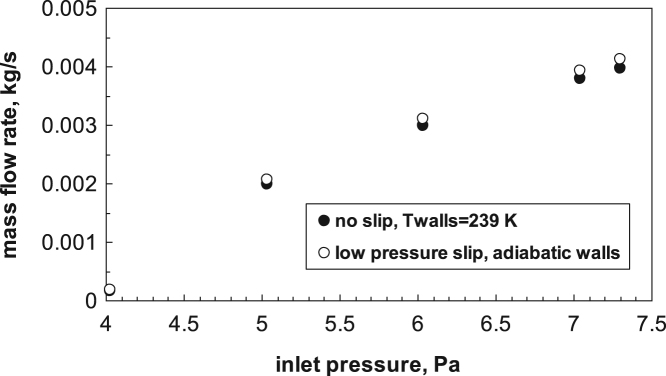
Mass flow in a duct with disk valve, as a function of the inlet pressure, for a constant outlet pressure. Two different boundary conditions are considered.

Fig. 4 shows that if the inlet pressure is increased, keeping constant the outlet pressure, the mass flow increases, and keep increasing also after that choked conditions are obtained; the data refer to subcritical flow conditions, with an outlet pressure close to 4 Pa. In the example the influence of different boundary conditions is also shown; for a DN 700 valve the Knudsen number is sufficiently low to make acceptable the no-slip assumption, thus the slight effect is mainly due to the influence of temperature at the wall. Graphic correlations shown in the following have been developed for adiabatic wall and no-slip conditions.

#### 2.2.4 Simulation of complete small scale equipment

The mesh used for the CFD simulations is made up of 619,401 cells in the case of the straight entrance and of 623,671 cells in the case of the rounded entrance. The incondensable gas pipe is not meshed, and its first section is modelled as the inert gas exit from the condenser.

The water vapor deposition in the condenser is modelled as a finite rate process taking place on the condenser refrigerated walls (its lateral walls); further details can be found in Ref. [6]. The two chemical species (water vapor and nitrogen) enter the chamber through uniform sources placed on the upper side of four slabs representing the layers of vials, where the inert gas represents the 5% of the overall entering mass flow. In the base case the sublimation rate considered is 1 kg h^−1^ m^−2^, with an interface product temperature of 239 K, and the pressure of 4 Pa is imposed at the condenser exit (at the entrance of the incondensable gas pipe), modelled as a pressure-outlet. The low-pressure-boundary slip option is activated in Fluent laminar model, considering the low pressure values and the small duct size.

#### 2.2.5 Mushroom valve fluid dynamics

The duct inlet is modelled as a mass flow inlet, imposing the inlet mass flow; the stagnation temperature must also be imposed, and has been taken equal to 239 K: it must be evidenced that differently from previous cases (pieces of duct, empty or with the butterfly valve), where the stagnation temperature to be fixed at the inlet was calculated for the different flow rates in such a way that the static temperature was always the same, here the same value of stagnation temperature is considered for the different cases, and as a consequence the static temperature may change at the inlet (the variation is of about 5 K between the lowest and the highest flow rate considered). The choice of constant stagnation temperature allows having the same static temperature in correspondence of the center of the disk, where the flow is stopped.

The outlet section, a plane positioned in the vessel behind the mushroom disk, is modelled as a pressure outlet; the outlet pressure is set equal to 4 Pa in all cases. It can be reminded that practically the total pressure is constant in a condenser, as the reduction in partial pressure of water vapor is compensated by the increase in the partial pressure of inert gas. The low pressure boundary slip conditions have been adopted; the walls of duct and vessel are modelled as adiabatic surfaces and radiation is neglected.

### 2.3 Analysis of the Knudsen number

The evaluation of the Knudsen number, defined in Eq. (3)

(3) $$Kn=\frac{\lambda }{r}$$

is necessary in order to assess the validity of the continuum approach; this is very important in the freeze-drying chamber since the governing equations are often pushed to their limit of applicability under common operating conditions, possibly resulting in an over-estimation of pressure drops and fluid velocities [1].

If *Kn* is very small (*Kn*<0.01) the gas flow is in the continuum regime, and the standard continuity and momentum balance equations can be used, while when *Kn* is large (*Kn*>1) the gas is in the so-called molecule regime and the BE has to be solved instead. If the mean free path of the gas molecules is neither very large nor very small, as compared to the macroscopic length-scale of the flow, the system is under the so-called transitional regime. In this case the boundary conditions for the governing equations are modified to take into account for velocity slip and temperature jump at the walls [7]. This is needed as the gas-phase velocity at solid surfaces differs from the velocity at which the wall moves and the gas temperature at the surface differs from the wall temperature.

For the cases reported in Table 2, the parameter *r* can be considered the characteristic dimension of the system. In order to evaluate the dependence of the Knudsen number on the clearance *r* it is necessary to evaluate the mean free path (*λ*) of the vapor molecules. From the kinetic gas theory, the mean free path can be written as in the following equation:

(4) $$\lambda \;=\frac{1}{\sqrt{2}\pi d^{2}}\frac{k_{B}T}{P}$$

where *k*_*B*_ is the Boltzmann constant, *T* is the temperature, *d* is the Lennard-Jones characteristic length of the molecule and *P* is the absolute pressure. It can be observed that *λ* is proportional to the temperature and inversely proportional to the pressure so, if the temperature is maintained constant, decreasing the pressure value the mean free path increases.

**Fig. 5:**
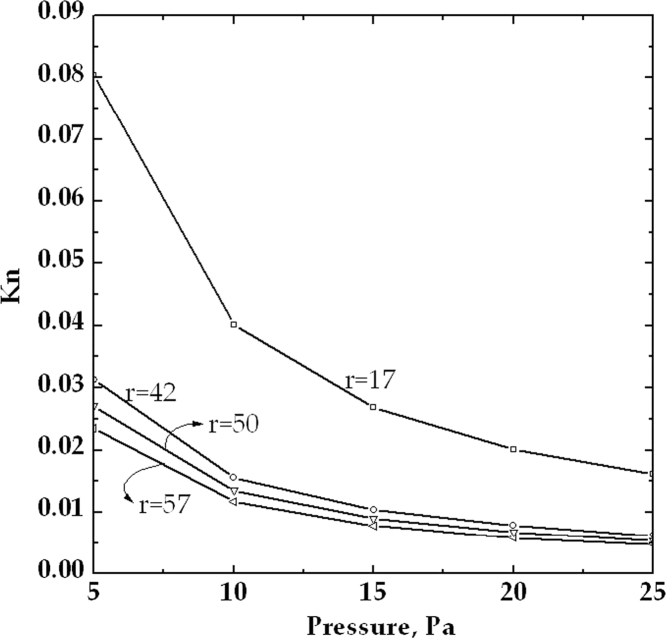
Dependence of the Knudsen number on the pressure calculated with 4 different clearances between the shelves and the product. The free distances shown correspond respectively to case S2 (*r*=17 mm), L4 (*r*=42 mm), L3 (*r*=50 mm), S1 and L2 (*r*=57 mm).

In Fig. 5 the *Kn* number depending on *r* has been reported for some of the cases investigated. The Knudsen number decreases if either the distance between the shelves or the pressure is increased.

The data confirm that, with the conditions considered, the regime is generally laminar, thus the simulations give reliable results, but moving to smaller free distances, at the lowest pressure, it is possible to enter in the transition regime. In particular, for very low values of *r* pure viscous flow (with *Kn*<0.01) occurs only at higher pressure; for higher clearance values the pressure can reach smaller values but also for this condition below 10 Pa the transitional regime for the vapor flow occurs.

## 3 Data analysis

### 3.1 Fluid dynamics in the pilot scale apparatus. Influence of clearance and duct position on flow field and pressure distribution

**Fig. 6:**
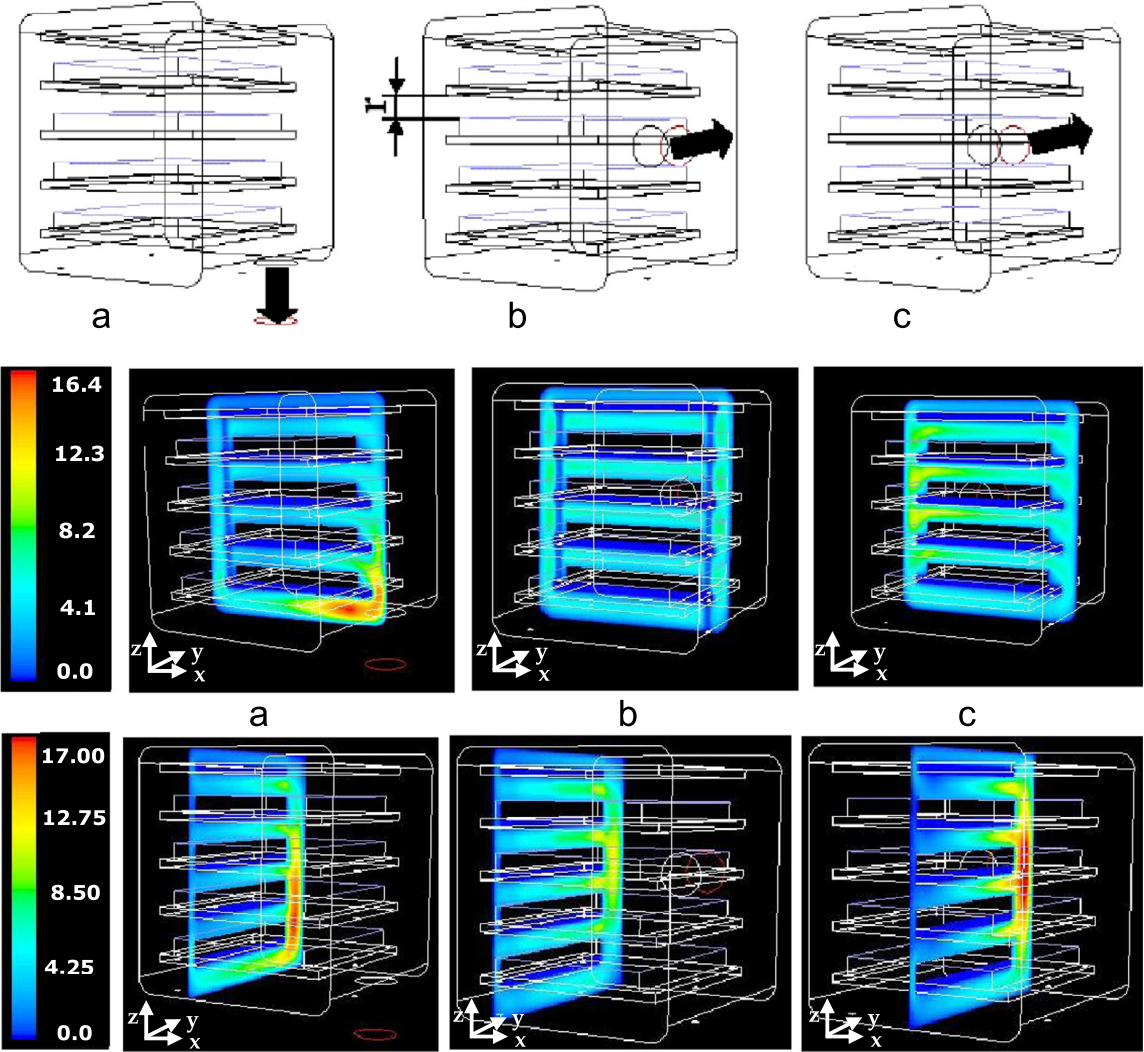
Upper graphs: 3D representation of the small freeze-dryer chamber with the duct in different positions: bottom (a), in the centre of the rear wall (b) and on a side of the rear wall (c). The slab that represents the volume occupied by trays or vials is shown, and the size of the free clearance *r*, is evidenced. Velocity magnitude (m/s) plotted on *x–z* plane (middle graphs) and velocity magnitude (m/s) plotted on *y–z* plane for the S1 case (*r*=57 mm) for the three configurations; a mass flow rate equal to 4.5·10^−5^ kg s^−1^, and outlet pressure 10 Pa, corresponding to typical values experimentally measured in lab operation has been considered in this case.

In Fig. 6 the contour plots of the velocity magnitude are reported on two different planes for all the investigated positions of the duct; the *x–z* plane is positioned perfectly in the middle of the chamber, while the position of the *y–z* plane has been chosen in order to keep the same distance from the duct and the plane for all the configurations. For these contour plots it can be observed that the maximum of the velocity is reached near the duct; moreover, when the duct is positioned at the bottom of the chamber, stronger velocity gradients are observed.

**Fig. 7:**
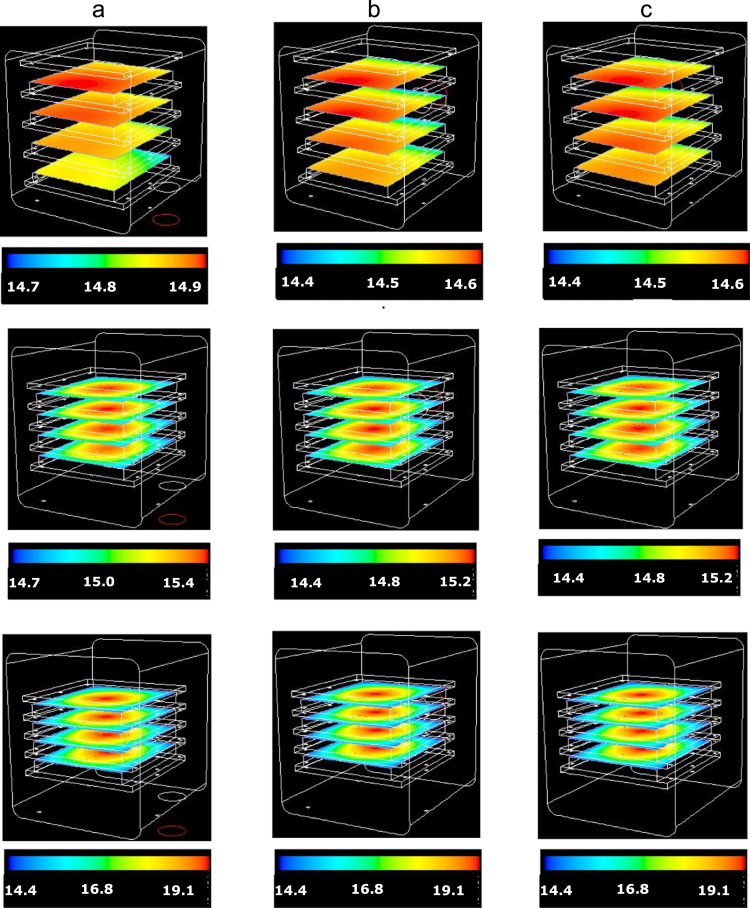
Contour plots of the absolute pressure (in Pa) over the vapour sources when the duct is positioned at the bottom of the chamber (a), at the centre of the rear wall (b) and on the side of the rear wall (c), for the three shelf clearances considered (from top to bottom, cases S1, S2 and S3, corresponding to *r*=57, 17 and 7 mm respectively).

Fig. 7 shows the pressure distribution over the shelves for different duct positions and shelf clearances (the two ones with the duct on the rear wall are very similar).

### 3.2 Temperature distribution in the chamber. Influence of inter-shelf clearance

**Fig. 8:**
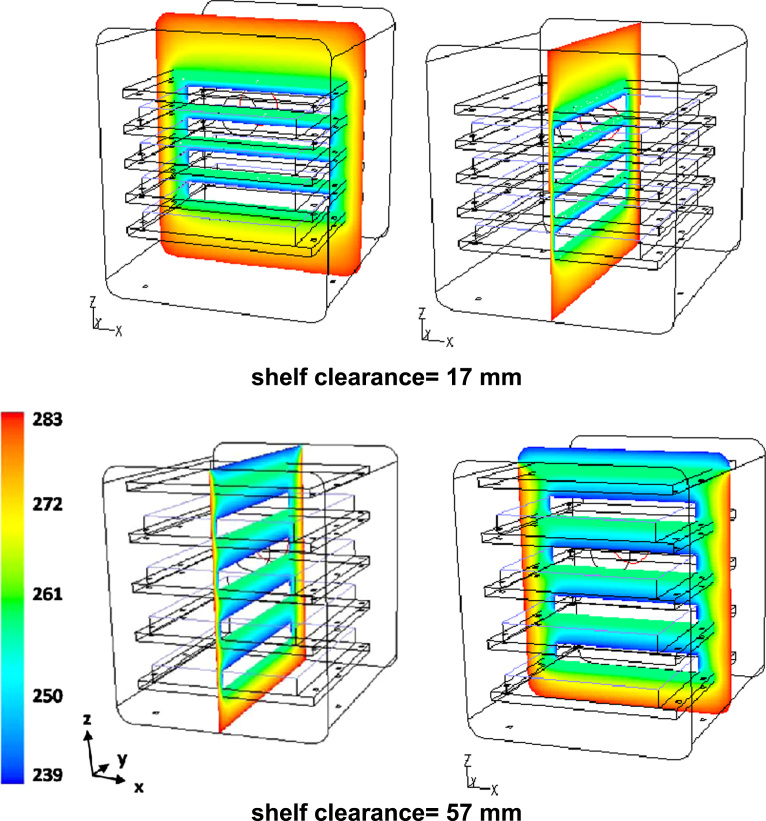
Contours of the static temperature in the small-scale chamber; configuration c) in Fig. 3 (duct on a side of the rear wall). Two different clearances are considered.

Fig. 8 shows the temperature distribution on two different orthogonal planes positioned in the middle of the chamber; no appreciable differences are observable for these clearances between the case with slip and no-slip boundary conditions (data not shown). In the upper graphs the walls are all at 283 K and it can be seen that the vapor in the outer clearance zone is at higher temperature and close to the wall temperature, but the gas temperature in the zone between the shelves is determined mainly by the sublimation process (temperatures are in the range 240–260 K, with the product at 239 K).

Changing the shelf clearance, the situation remains similar. In the bottom graphs a different boundary condition is adopted for the top surface (an adiabatic wall is considered).

The area-weighted average temperature calculated on the two planes shown resulted to be 266.59 and 258.71 K in the two cases (266.35 and 258.56 K respectively with slip boundary conditions).

### 3.3 Fluid dynamics in the large scale apparatus. Influence of clearance on flow field and pressure distribution

**Fig. 9:**
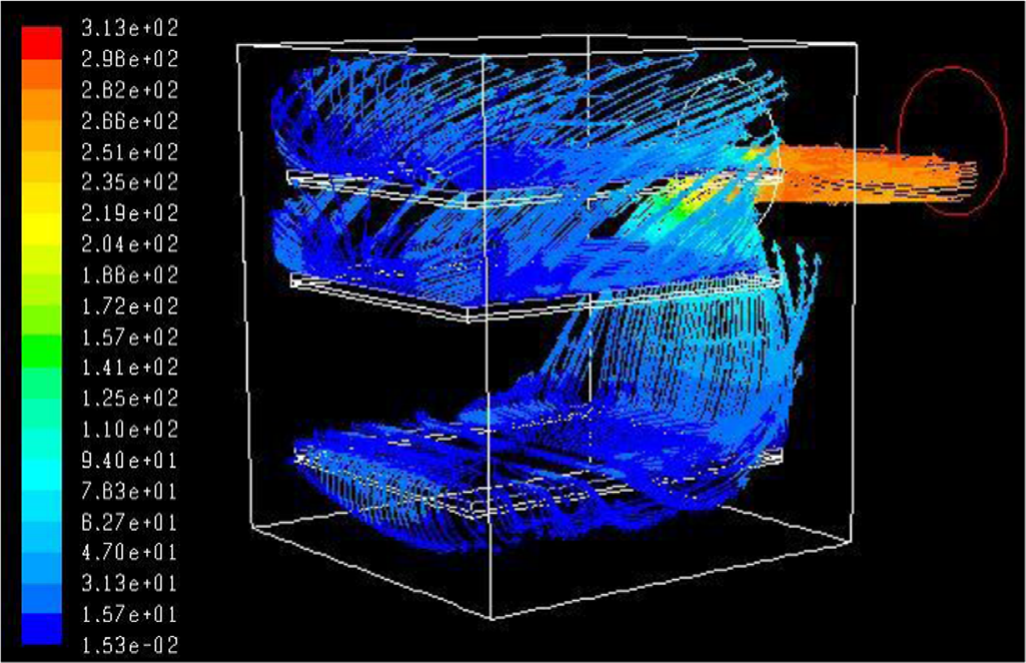
Examples of the streaklines of the vapor flowing out of the large scale chamber (velocity is in m/s): for sake of clarity only the flow coming from the 1st, 8th and 12th of the 14 shelves (corresponding to a distance *h* equal to 110 mm and an effective clearance *r*=67 mm, configuration L1) is shown.

In order to give a general idea of the fluid dynamics in the chamber, the streaklines of the vapor flowing from the shelves to the duct connecting the drying chamber to the condenser can be used to visualize the fluid trajectory in the drying chamber. An example is given in Fig. 9: as it is possible to see from the velocity vectors, the vapor sublimating from the vials is forced to flow toward the edge of the shelf and, from there, it directly goes into the duct positioned on the rear wall, to be then collected in the condenser.

**Fig. 10:**
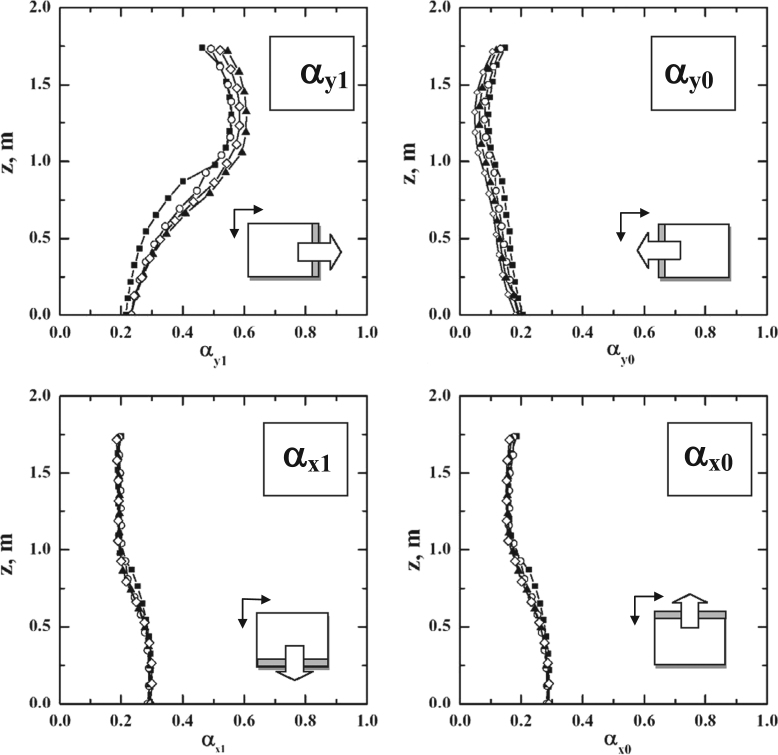
Coefficients α_*y*1_, α_*y*0_, α_*x*1_ and α_*x*0_ representing for each shelf the fraction of vapour flowing out of the selected side (see the sketch in the lower right corner, and consider that the side along *y* is slightly longer). Graphs as a function of the *z*-coordinate for the four configurations of the large scale freeze-dryer, with 1 kg h^−1^ m^−2^ sublimation flux: ◊ , L1; ▲, L2; ○, L3; ■, L4.

To design purposes, it can be useful to quantitatively evaluate the flow distribution between the different sides of the shelves, to verify if the clearance between the shelf-pack and the walls is appropriate; the geometrical solution which gives a uniform flux through the lateral zones of the shelves will assure the minimum pressure drop in the chamber. In Fig. 10 the coefficients α_*xi*_ and α_*yi*_ as functions of the *z* position are shown (0 add 1 in the pedex refer respectively to the side closer to and farer from the axis origin. These coefficients represent for each shelf the fraction of vapor flowing out of the selected side and are calculated as the ratio between the mass flow rate *m*_*xi*_, *m*_*yi*_ and the total mass flow rate of vapor; these values have been obtained by integrating the mass flux obtained by CFD simulations over the surfaces that delimit the shelf volume, and can be useful if the pressure drop over different shelves has to be compared. Vapor rate fraction coefficients sum to unity:

(5) $$\alpha_{x0}+\alpha_{x1}+\alpha_{y0}+\alpha_{y1}=1$$

It is interesting to observe that the coefficients of the vapor distribution relative to the *x*-direction are not affected by the number of shelves, while this effect exists, even if it is weak, in the *y*-direction; moreover, for the plates at the bottom of the chamber, far from the duct, an equivalent vapor distribution through the four sides of the shelves is observed (it must be remembered that one side is 20% longer than the other one); moving up, the value of α_*y*1_ increases and reaches the maximum value of 60% for the shelves close to the duct.

In the apparatus considered the duct is on one side, thus pressure profile over the shelves is not symmetric, and changes with the shelf position (the farer from the duct, the more symmetric they are: see also Figs. 4 and 6 in Ref. [1]). In Fig. 5 in Ref. [1] *y_max_*, the *y*-coordinate of the maximum pressure value for each shelf, and the corresponding maximum pressure difference over the shelf are reported for all the configurations, as a function of the vertical position of the shelf.

**Fig. 11:**
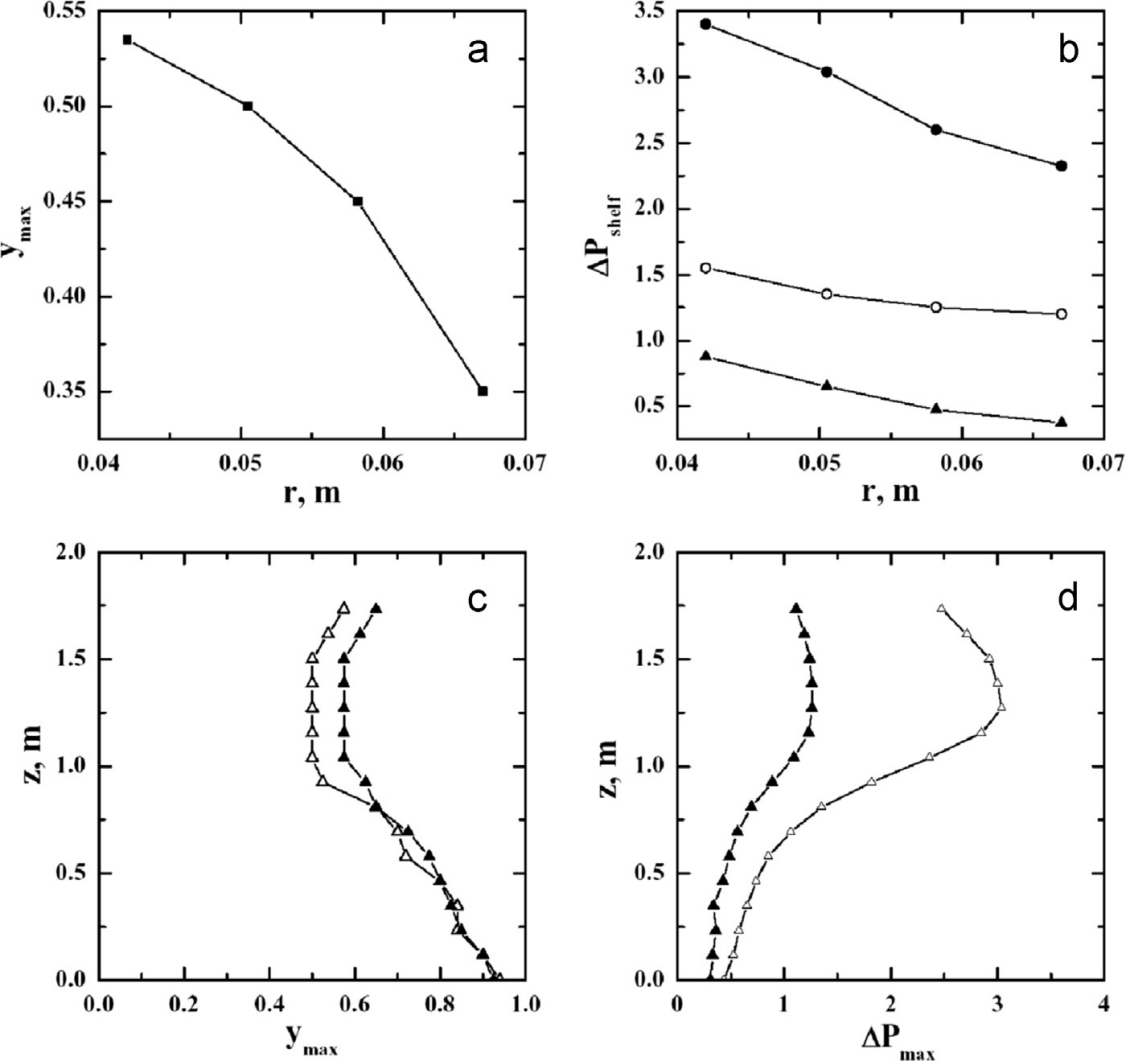
Influence of the clearance, *r*, on the location of maximum pressure, *y_max_*, for the shelves at the level of the duct (a), and on Δ*P_max_*, for three different shelves, numbered from bottom (b): ▲, third shelf; ○, sixth shelf; ●, shelf close to the duct. On the lower graphs, the variation of *y*_max_ (c) and Δ*P_max_* (d), with the *z*-coordinate for the L3 configurations at two different sublimation fluxes: Δ, 1 kg h^−1^ m^−2^; ▲, 0.5 kg h^−1^ m^−2^.

The dependence of these quantities on the inter-shelf clearance is explicitly shown in Fig. 11. In particular, in Fig. 11a it is shown the variation of the location of the maximum pressure in the shelves at the level of the duct. The maximum pressure drop (Δ*P_max_*) over three different shelves is plotted as a function of clearance in Fig. 11b (at maximum sublimation flux of 1 kg h^−1^ m^−2^). Δ*P_max_* decreases when clearance *r* increases; it can be noted that the dependence of Δ*P_max_* on *r* is different for the different shelves. Very important is the effect of the sublimation rate. In Fig. 11c–d the effect of the mass flow rate on *y_max_* and Δ*P_max_* is evidenced for the case L3 (16 shelves). The major effect of the mass flow rate is to increase Δ*P_max_*, but this increase is larger for the shelves close to the duct.

**Fig. 12:**
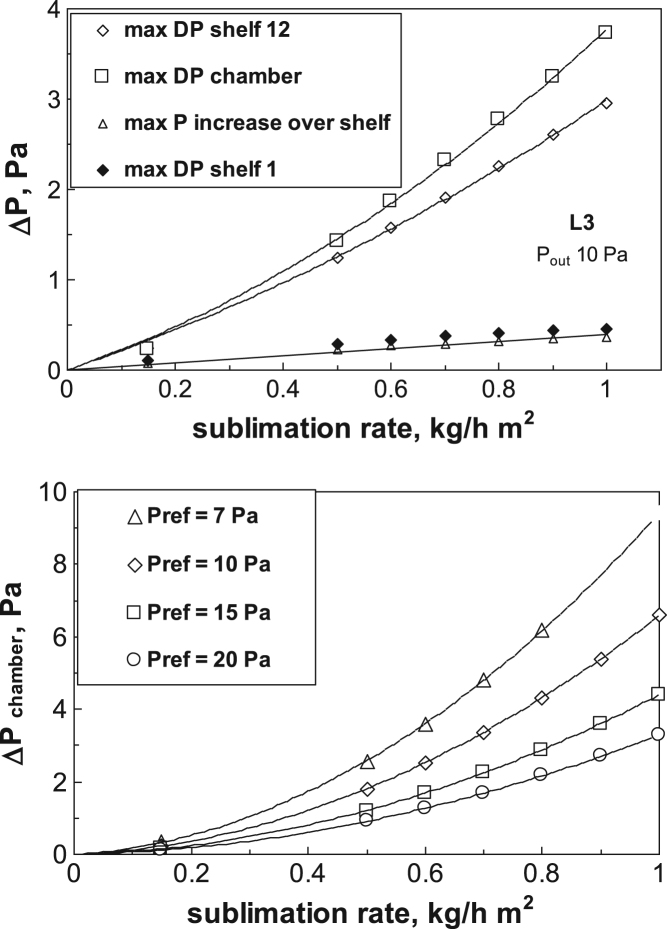
Upper graph: comparison of maximum pressure variations over different shelves (shelf 1 and 12 are selected) and in chamber, as a function of the sublimation rate. The maximum pressure increase with respect to reference pressure (that occurs on the lowest shelf) is also shown. Lower graph: pressure drop in the chamber as a function of sublimation rate and reference pressure. L3 configuration.

In Fig. 12 (upper graph) the values of the pressure differences over the shelves and in the chamber are compared (for the L3 configuration, with 16 shelves). It can be noted that in the central zone of the shelves, pressure values higher than the reference one can be observed: the maximum value occurs in the lowest shelf, less affected by the duct, but the maximum pressure increase, for the configuration shown is in any case very small, lower than 0.5 Pa. In Fig. 12 (lower graph) The effect of the chamber reference pressure is shown.

### 3.4 Distribution of inert gas in the drying chamber

#### 3.4.1 High flow rate regime for the injected inert gas

The data presented have been obtained for a pilot scale drying chamber where the inert gas inlet has been supposed positioned in the top corner of one of the lateral walls of the chamber. Results concerning the range 1.20·10^−8^–1.20·10^−5^ kg s^−1^, which covers the values normally observed during primary drying, have been presented in Fig. 7 in Ref. [1]. In the secondary drying, in particular, much higher values can be measured, as the desorption flux of the solvent has significantly lower values.

**Fig. 13:**
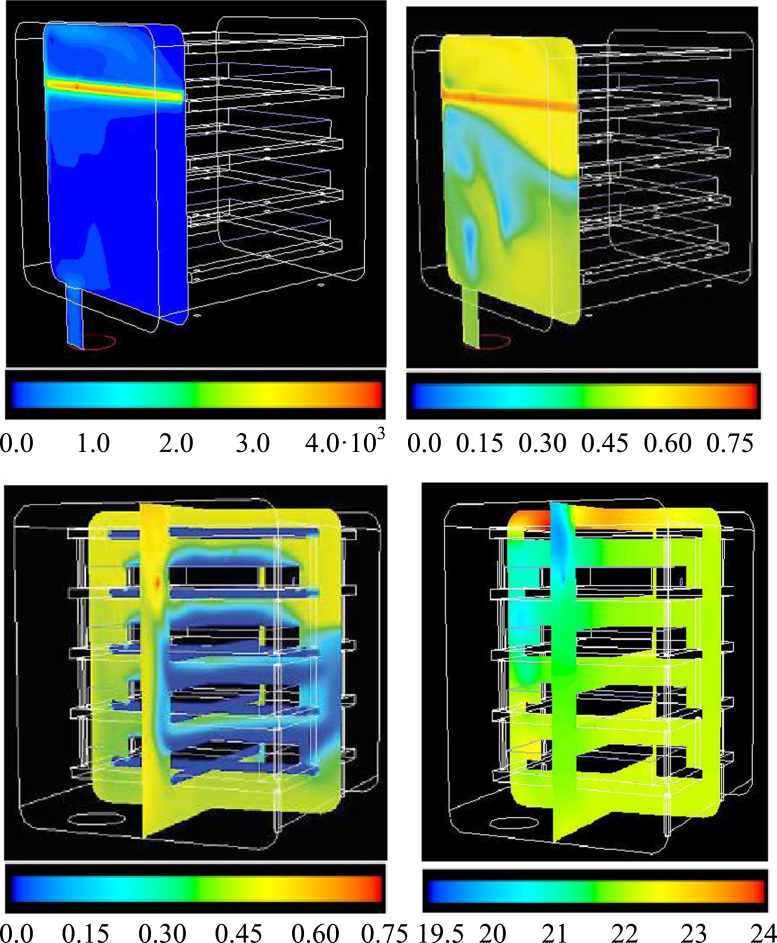
Inert gas mass fraction distribution in the small scale drying chamber for very high inlet flow rates (1.20·10^−4^ kg s^−1^). Outlet pressure=10 Pa; water sublimation rate 1 kg h^−1^ m^−2^. Velocity magnitude (upper left, m/s) and inert gas fraction (upper right, -) are shown on the plane of the inlet inert gas jet. In the bottom graphs the inert gas fraction (left, -) and the absolute pressure (right, Pa) are shown on two orthogonal middle planes. Red colour corresponds to maximum value for each case, yellow to 75%, green to 50%, light blue to 25% and dark blue to 0.

Contour plots (on the jet plane) of inert gas fraction and velocity magnitude for the highest inert gas bleed rate investigated (1.2·10^−4^ kg s^−1^) are shown in Fig. 13 (top graphs). In this case extremely high velocities are obtained, and the jet reaches the opposite wall generating local high pressure values (>50 Pa).

In Fig. 13 (bottom left) it is evidenced that, in the clearance between the shelves, the inert gas mass fraction remains low, while with this configuration higher values are observed in the lateral space. In the bottom right image the pressure is shown in two cross planes, where it ranges from about 20 (blue) to 24 (red) Pa; for comparison, it can be mentioned that, without inert gas injection, a quite uniform 14 Pa value is obtained.

#### 3.4.2 Influence of inert gas fraction on the heat transfer coefficient

Even if the average value of the inert gas fraction is low (but not null) during primary drying with gas-bleeding pressure control, depending on the geometric configuration, locally relative high values of inert gas fraction can occur. As the heath transfer coefficient between shelf and vials depends on the gas composition, this fact can affect the uniformity of the batch. In any case the heat transfer during secondary drying will be strongly affected, and this must be taken into account in cycle design.

**Fig. 14:**
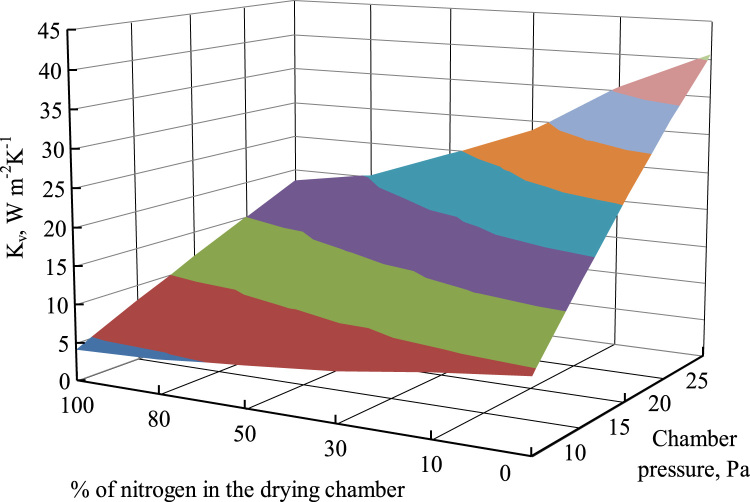
Overall heat transfer coefficient as a function of chamber pressure and gas composition (% of nitrogen); vial: internal diameter=14.25 mm, bottom thickness=0.7 mm, maximum bottom gap=0.4 mm.

An example of *K*_*v*_ calculated for various values of total chamber pressure and of nitrogen percentage in the drying chamber is shown in Fig. 14. The case study here analyzed is the freeze-drying of a 10% w/w sucrose aqueous solution in tubing vials. Values shown in the figure have been calculated for a vial having an internal diameter of 14.25 mm, with a wall thickness of 1 mm, a bottom thickness of 0.7 mm, and the maximum gap between the bottom and the shelf of 0.4 mm; the shelf temperature is assumed to be equal to 253 K. The operating conditions used for the calculations are: *P*_*c*_=10 Pa, *T*_*shelf*_=−10 °C. It can be seen that the higher is the nitrogen percentage in the drying chamber, the lower is the value of *K*_*v*_.

**Fig. 15:**
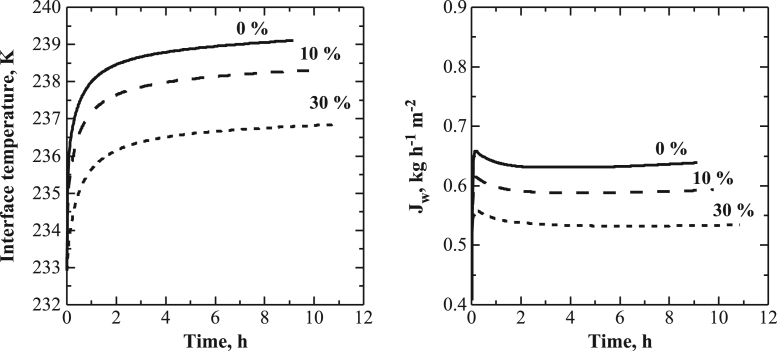
Evolution of interface temperature and sublimation flux, *J*_*w*_, during primary drying, depending on the local gas composition in the chamber (% of inert gas fraction). 1 ml of 10% w/w sucrose solution in vial (14.25 mm internal diameter); chamber pressure = 10 Pa, *T*_*shelf*_ =−10 °C.

An example of the influence of the local inert gas fraction on the drying behavior of the product in vials is shown in Fig. 15. A relatively wide range is considered for the nitrogen fraction, for a sensitivity analysis; the pure water vapor case can be representative of processes where chamber pressure is maintained acting on the vacuum pump, neglecting the leakages from the environment.

### 3.5 Transient chamber modelling

The details of the conditions of all the simulations carried out are given in Table 3. In the simulations D1-D6 all the shelves are considered loaded with product and then all the vapor sources are considered operating; in these cases the flow field in the drying chamber is the same obtained with the steady-state simulations. For the simulations D7 and D12 only the shelf at the top of the chamber (*ss*_4_) has been considered operating; in these cases new steady-state simulations are carried out before adding the scalar because the flow field of the previous simulation is no more representative for this system.

**Table 3:** Scheme of the scalar transient simulations.

**Case**	Sublimating source	Mass flux, kg m^−2^ h^−1^	Disturbance source	Diffusivity accounted
D1	all	1	all	–
D2	all	1	all	yes
D3	all	1	*ss*_1_	–
D4	all	1	*ss*_3_	–
D5	all	1	*ss*_3_	yes
D6	all	1	*ss*_4_	–
D7	*ss*_4_	1	*ss*_4_	–
D8	all	0.5	all	–
D9	all	0.1	all	–
D10	all	0.1	all	yes
D11	all	0.1	*ss*_3_	–
D12	*ss*_4_	0.1	*ss*_4_	–

Several different sources of disturb have been considered: on all the sublimation surfaces (simulations D1, D2, D8, D9, D10, D13, D14), only on a specific shelf (*ss*_1_ for D3, *ss*_3_ for D4, D5 and D11, *ss*_4_ for D6, D7 and D12). In some simulations (D2, D5 and D10) also the scalar diffusivity has been taken into account (considered equal to the kinematic viscosity).

**Fig. 16:**
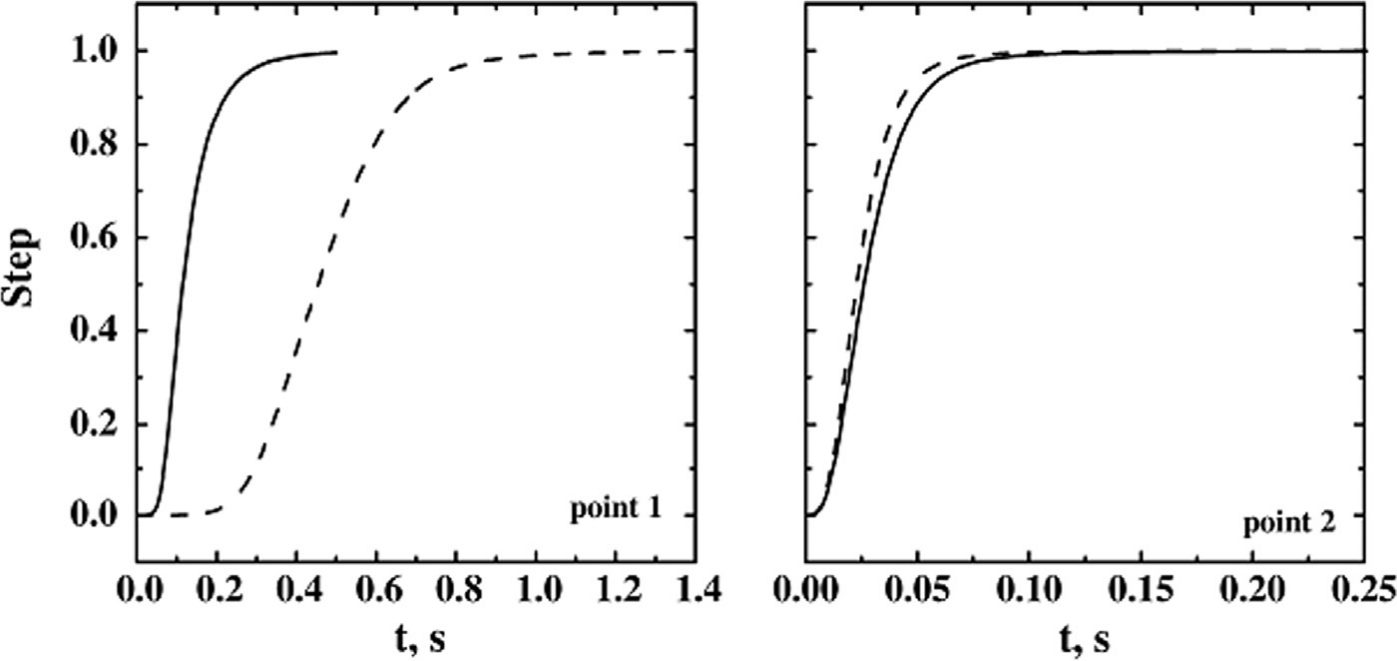
Dynamic response of the small pilot freeze-dryer to disturbances in the sublimation rate of different shelves; the location of the virtual measuring devices is shown in Fig. 1. Response in the different detection points (point 1 and 2 are shown): the cases of a single loaded shelf (- - -, *ss*_4_, D7) and of fully loaded system (−−, D1), with disturbance applied to all the shelves, are compared. *J*_*w*_=1 kg h^−1^ m^−2^.

In Fig. 16 cases D1 and D7 are compared; in these simulations the full load and the partial load (but on the top shelf, close to the virtual detectors) of the chamber are compared. In the graphs, the dynamics of the system in different positions is shown. The dynamics is quite similar when the response is detected in points 2, 3 and 4 (thus only point 2 is shown for brevity); this aspect is very important for the sensor response interpretation. In point 1 the delay for the partial-load system (D7) is higher than for the fully loaded system (please note that the time scale on the axis is different). This results can be explained comparing the flow-field of the two systems, in fact the velocity in D1 is higher than in D7.

For the sensor response interpretation it is important that differences in the sensor response are small in full and partial-load cases; for sensor location 1 delays are slightly different (even if differences are relatively small at high sublimation rate). But it must be underlined that this consideration is true only if the load is on the upper shelf *ss*_4_; in fact, in case D4 no signal can be detected in points 2, 3 and 4.

**Fig. 17:**
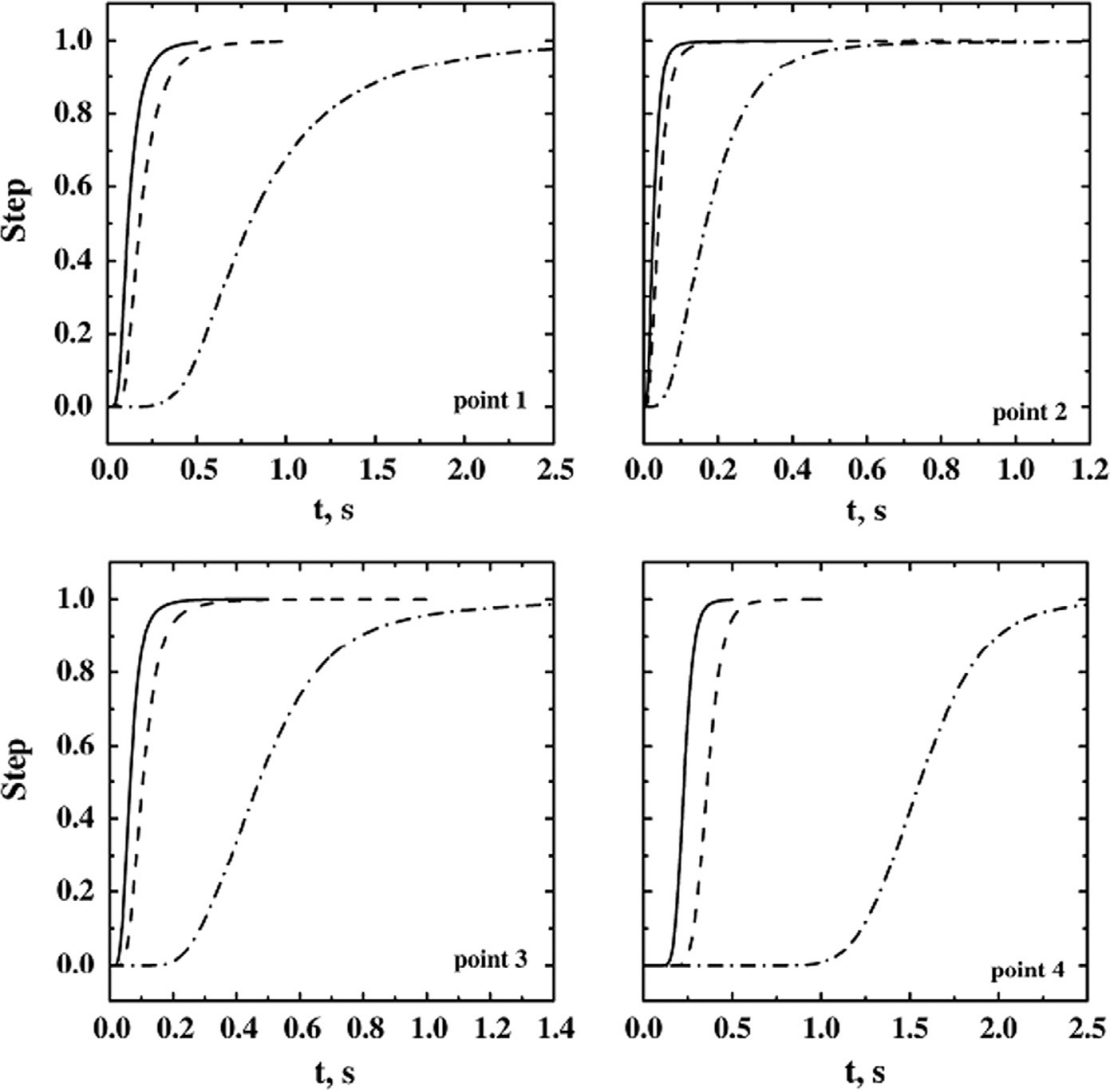
Response in the four detection points for the fully loaded system, with disturbance applied to all the shelves. Response for different sublimation rates are compared: —, *J*_*w*_=1 kg h^−1^ m^−2^ (D1); - - -, *J*_*w*_=0.5 kg h^−1^ m^−2^ (D8); −·−, *J*_*w*_=0.1 kg h^−1^ m^−2^ (D9).

The effect of the sublimation flow rate on the dynamic response of the system has been assessed with the simulations D1, D8 and D9 for the full-load system. In Fig. 17 the results of these simulations are reported. Decreasing the sublimation rate both the response delay and the rise time increase.

**Fig. 18:**
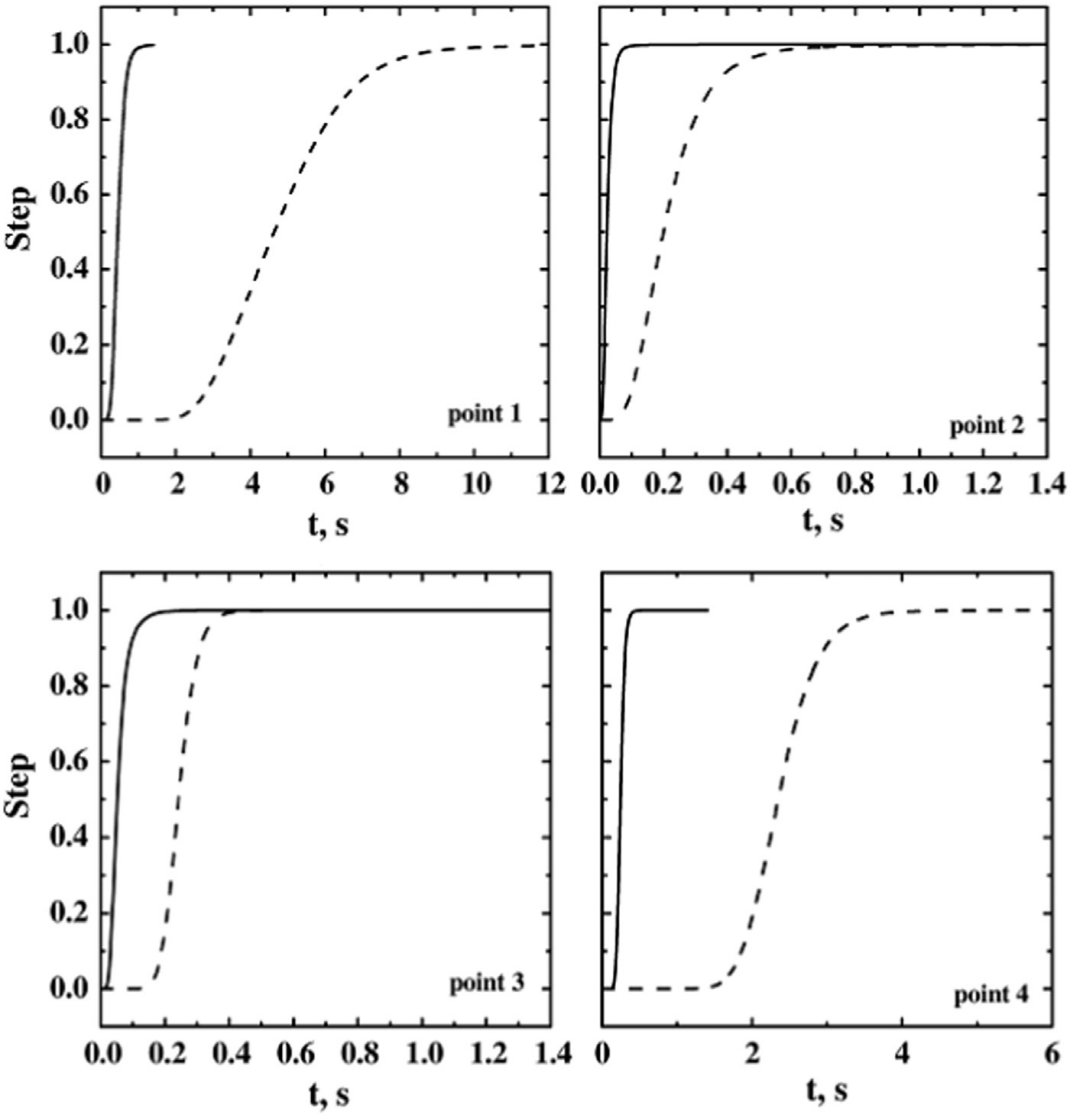
Response in the four detection points for the single shelf loaded system (*ss*_4_,), with different sublimation rates: —, *J*_*w*_=1 kg h^−1^ m^−2^ (D7); - - -, *J*_*w*_=0.1 kg h^−1^ m^−2^ (D12).

**Fig. 19:**
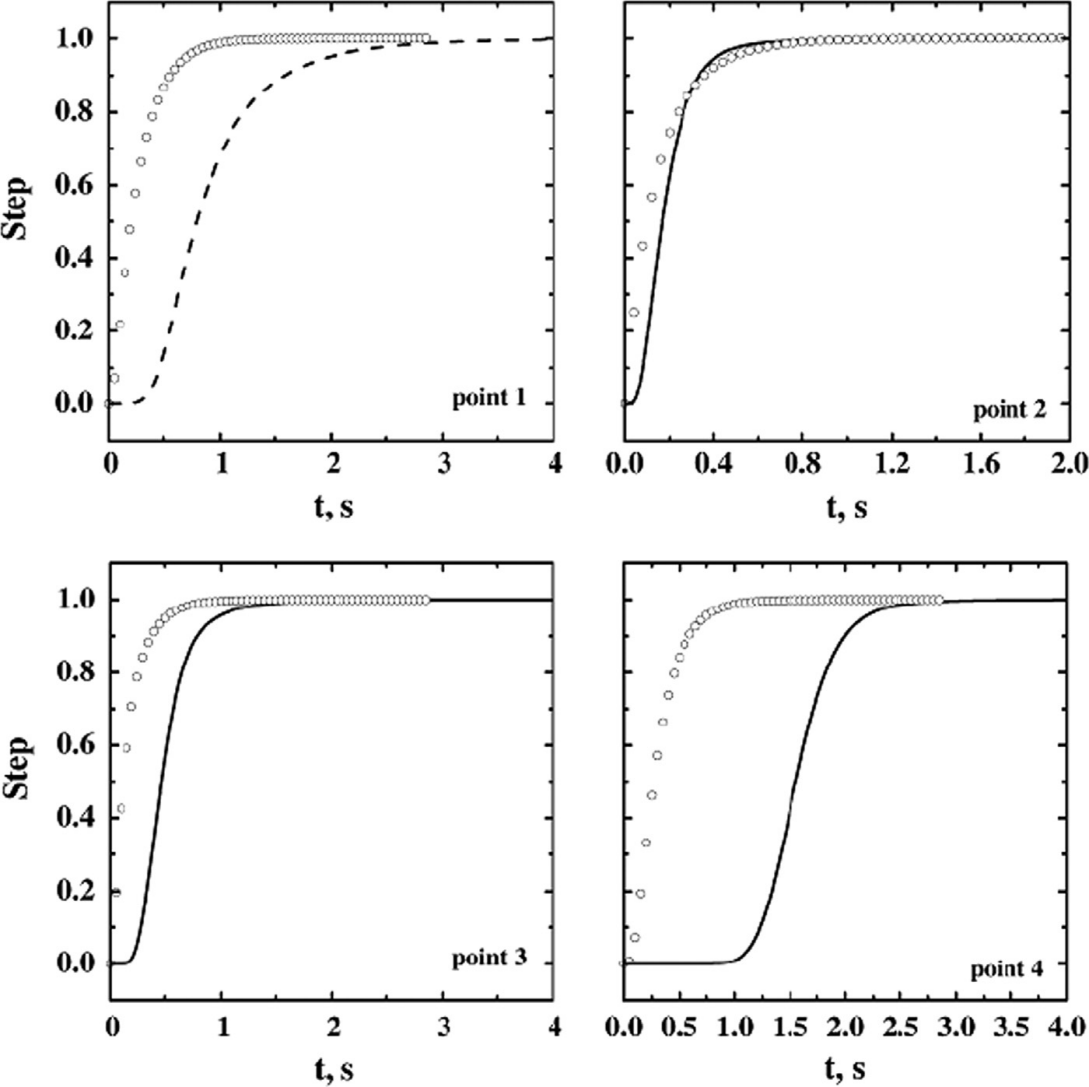
Response in the four detection points, without species diffusivity (simulation D9, line) and with species diffusivity (D10, symbols); fully loaded system, low sublimation rate, *J*_*w*_=0.1 kg h^−1^ m^−2^.

Similar results were obtained with partial load (simulations D7 and D12) (see Fig. 18). The delay increases significantly at low sublimation rates, but at very low mass flow rate the effect of scalar diffusivity becomes important. In Fig. 19 the dynamic response calculated taking or not the diffusivity into account is compared. When the scalar diffusivity is accounted for, the system response is faster; moreover the response detected in the four different positions is quite similar. Further data and discussion can be found in Ref. [1].

### 3.6 Maximum flow in ducts: dependence on duct length, diameter and pressure

The influence of the duct length on the maximum available vapor mass flow has been investigated considering different L/D ratios: the range 1–5 is the one of largest interest, and in literature data up to now available are limited to this range, but here data are presented in an extended range from 1.2 to 50, to allow checking the validity of the equivalent length concept for valves (see Ref. [2]). The simulations have been carried out for DN 700, but the results have been generalized using the mass flux; eventually a correction factor can be applied for smaller ducts.

All the plots refer to surface average pressures.

**Fig. 20:**
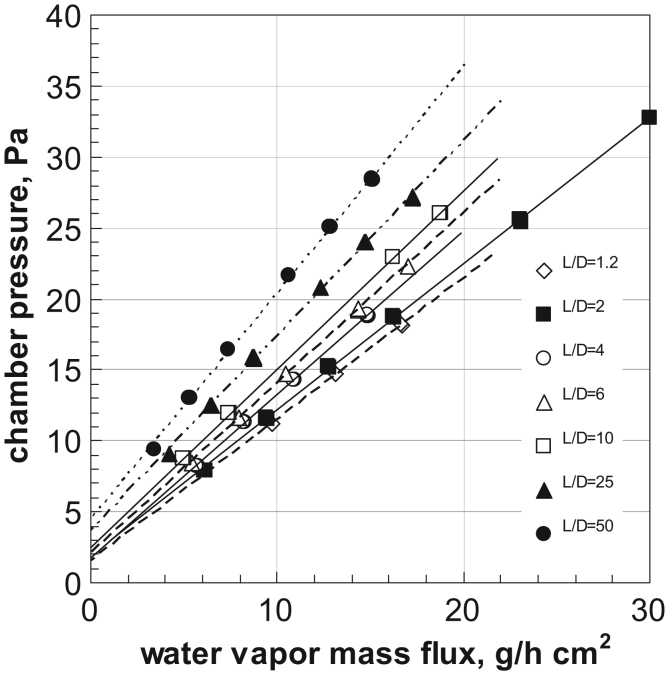
Critical mass flux as a function of the chamber pressure for different *L*/*D* ratios. CFD calculations carried out with DN 700; surface average pressure, no-slip and adiabatic wall boundary conditions.

The results obtained are shown in Fig. 20 (data at *L*/*D*=5 and 7 have been omitted for sake of clarity). It is evident that a linear relationship exists between the inlet (chamber) pressure and the critical mass flow density.

The relationship between pressure drop and flow rate, and the maximum allowed flow in choked conditions, depends on the characteristics of the compressible gas, that is on the inert gas fraction in the water-nitrogen mixture. Here some data obtained with “jet flow” calculation are presented in order to show which the influence of the different factors is. The jet flow depends on gas temperature and inlet Mach number (see [2]). Qualitatively the results for a water-nitrogen mixture are very similar to those obtained for pure water, but the total mass flux increases in presence of inert gas.

**Fig. 21:**
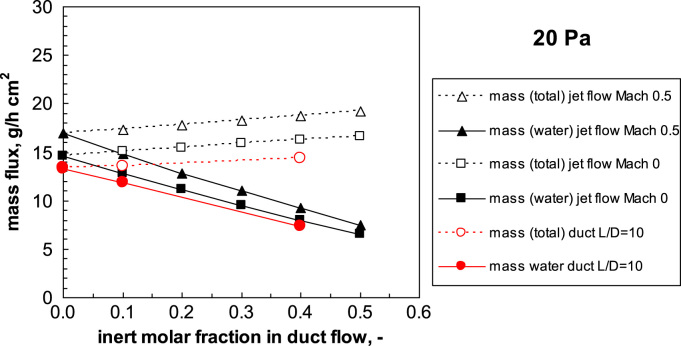
Calculation of critical mass flux as a function of the inert gas fraction at 20 Pa chamber pressure; jet flow calculation and CFD results for a duct with *L*/*D* = 10 are compared. Open symbols: total mass flux; filled symbols: water mass flux; in red (circles) the data referring to the duct calculated by CFD.

The critical mass flux increases with the initial Mach number, as can be seen in Fig. 21. The figure also shows how the critical flux increases progressively when the fraction of nitrogen gas increases; the molar flow rate is higher for pure water vapor and decreases when the inert gas fraction increases.

From the operating point of view, it is interesting to evaluate which is the influence of the inert gas on the mass flow rate of water that is possible to remove in the new conditions, as this is related directly to the sublimation rate in the drying chamber. Fig. 21 compares the total mass flux and the water mass flux as a function of the inert gas fraction in the mixture, at different Mach number, for a given chamber pressure (similar results have been observed for other pressures). Both quantities increase with the chamber pressure, but while the total mass flow rate increases with the inert gas fraction in the mixture, the water mass flow rate is reduced significantly, and more than proportionally.

In the jet flow calculations, the inlet Mach number, and thus the inlet kinetic energy of the flow, has been taken into account, as the critical flux is significantly affected by this variable; but while this is easy to estimate in case of flow through a hole, because it corresponds to the velocity in the vessel (and is generally close to zero), it is much more complicate to estimate it in a duct. But this is not really a problem, because the interest of the designer is in the reduction of the water mass flow that occurs in case inert gases are present. All the curves scale in the same way, thus the relative reduction in the water mass flux is the same independently of the inlet Mach number and chamber pressure.

If the behavior of straight ducts is analyzed, using CFD simulations, it appears that similar results are obtained (see Fig. 21). In this case the pressure at the inlet and outlet is set, thus the inlet Mach number is not a free variable, but is determined by the flow rate and the pressure drop in the duct. In the example a relatively long duct is considered; it can be noted that in this case the jet flow calculation significantly overestimates the mass flux, even if zero inlet velocity is considered.

### 3.7 Flow around butterfly valves

**Fig. 22:**
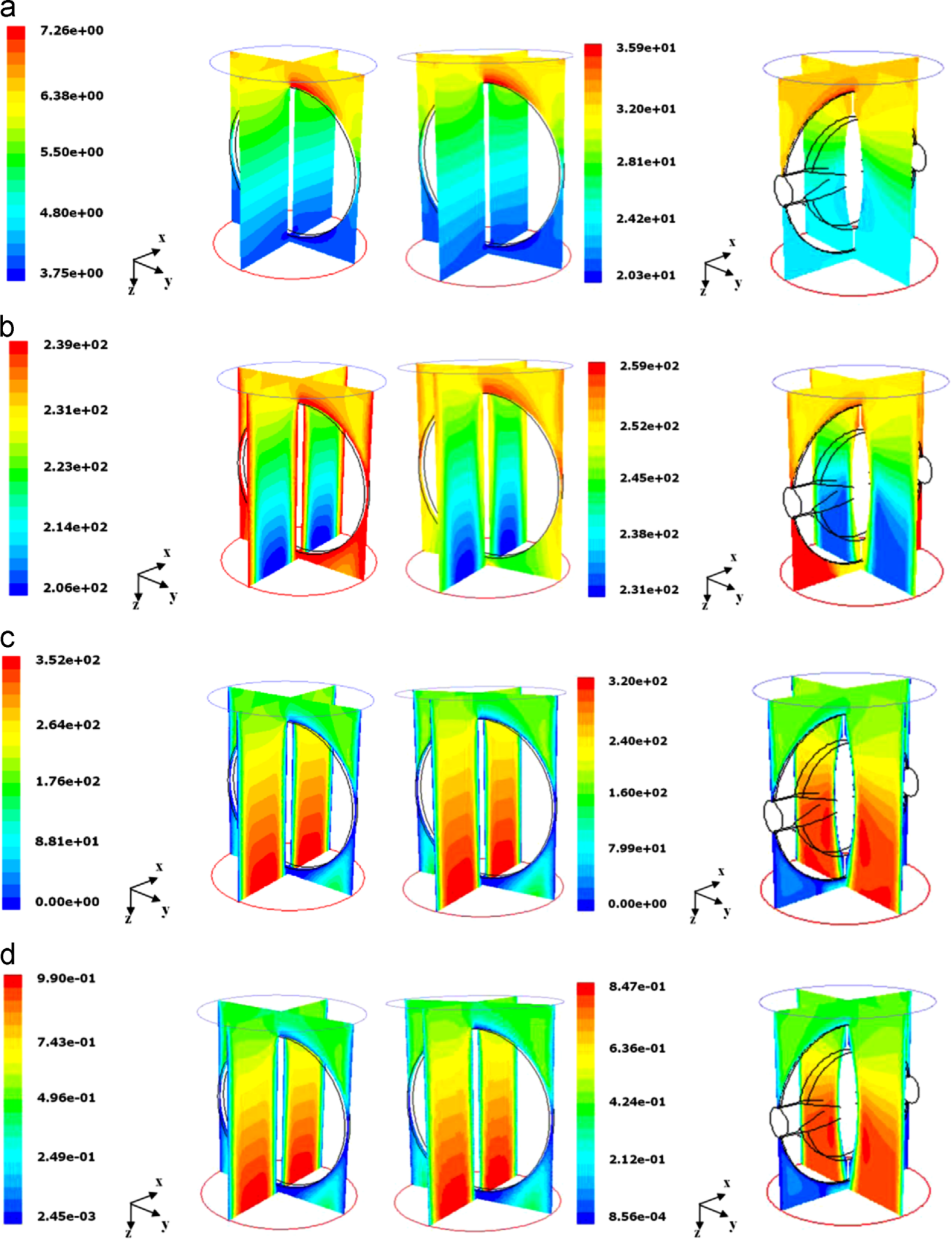
Butterfly valves; comparison of the flow with different boundary conditions and disk shapes. Left column: flat disk, low pressure slip and 239 K constant temperature; central column: flat disk, no-slip with adiabatic wall; right column, profiled disk, low pressure slip and adiabatic wall. From top to bottom, contours of: (a) absolute pressure (Pa); (b) static temperature (K); (c) velocity magnitude (m/s); (d) Mach number (dimensionless).

Fig. 22 (left and central columns) compares the flow obtained in the flat disk butterfly valve with the two different boundary conditions, showing at the same time the development of the pressure and velocity profiles in the two sections of the duct divided by the disk; the case shown corresponds to that of transonic flow: in fact, the Mach number approaches one at the exit.

It can be seen that in case of adiabatic flow the temperature decreases significantly, and the temperature of the wall (assumed equal to the inlet static temperature) has some influence on the developing of the velocity profiles and finally on the allowable mass flow. For a DN 700 valve the Knudsen number is sufficiently low to make acceptable the no-slip assumption, thus the slight effect is mainly due to the influence of temperature at the wall.

In the right hand column, the corresponding pressure, temperature, velocity and Mach number profiles obtained for the valve with profiled disk are shown.

### 3.8 Duct fluid dynamics in real apparatus

#### 3.8.1 Inlet effects in the large-scale apparatus

As concerns the large-scale apparatus, just a straight duct, with a *L*/*D*=2 has been considered.

**Fig. 23:**
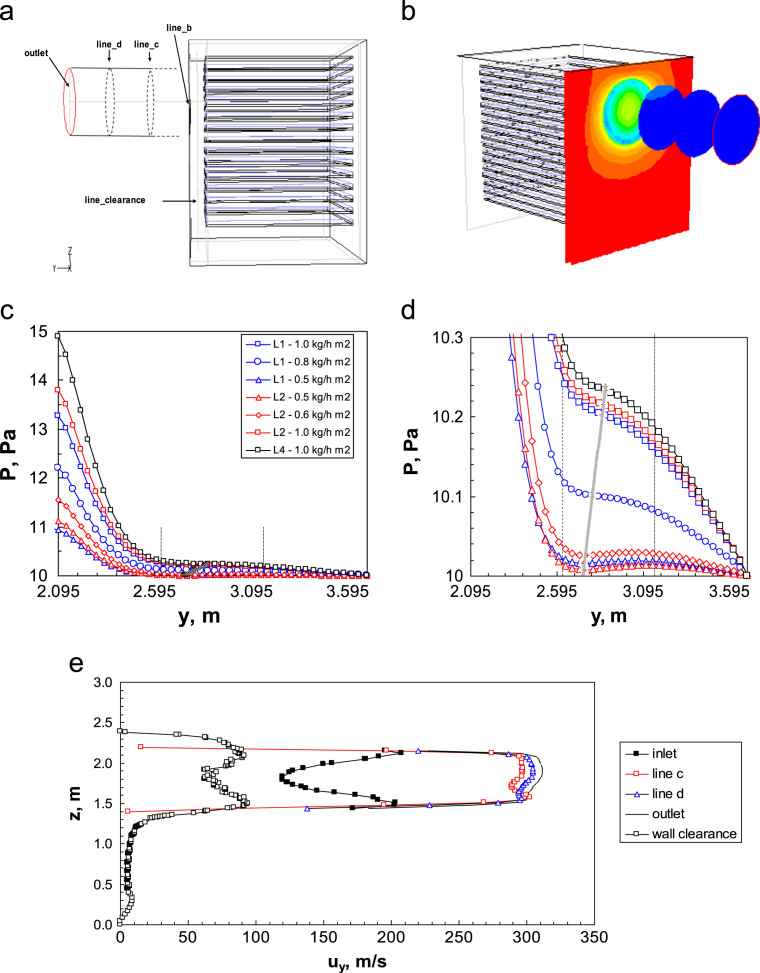
(a) Sketch of the large-scale industrial apparatus with the duct portion modelled; the position of the planes where the radial pressure and velocity profiles are shown is evidenced: line *b*, *y_duct_*=0; line *c*, y*_duct_*=533 mm; line *d*, *y_duct_*=1067 mm; outlet, *y_duct_*=1600 mm. (b) Pressure contour plots in the sections corresponding to the plane *b* (duct inlet), *c*, *d* and outlet; configuration L4, sublimation rate=1 kg h^−1^ m^−2^. Pressure values range from 10 Pa (blue) to 18.5 Pa (red). (c) Pressure profile along the duct axis, at different sublimation rates and for different configurations; the position corresponding to plane *c* (- - -) and *d* (−·−) is shown. *y*-axis coordinates are from the origin, including chamber. (d) Enlarged detail of previous figure, evidencing the zone where the sign of the pressure gradient derivative changes. The end of the inlet zone is evidenced by the thick grey line. (e) Radial velocity profiles in the planes selected along the duct and in the lateral clearance of the chamber (same condition as in b).

Fig. 23a shows a sketch of the apparatus, with the exact location of the planes where radial pressure and velocity profiles are given. Fig. 23b shows a 3D contour plot of the pressure profiles along the duct, showing the large gradients at the chamber-duct connection, and the non-uniformities still remaining in the duct (the radial pressure profile are shown in Fig. 6a in Ref. [2]).

Fig. 23c shows the pressure profile along the centerline of the duct, at different value of the mass flow rate (different configurations, with variable number of loaded shelves, and different sublimation rates have been considered). The end of the inlet zone is approximately at *y*/*D*=1, slightly affected by the mass flow rate as evidenced in Fig. 23d. It can be noted that a new inlet occurs, due to the development of the final parabolic profile.

Also the velocity profile is very different from the ideal one in a simple empty duct: Fig. 23e shows the radial velocity profile in the same planes of Fig. 23a and b (the zone close to the wall has been omitted for sake of clarity, but the slip effect is negligible). It can be seen that the profile has two lateral maximums, with the minimum on the centerline; this is a consequence of the particular velocity profile in the chamber clearance, but it becomes even more pronounced at the entrance plane where the maximum velocity almost doubles. It can be noted that in the following sections these strong differences disappear, but the profile remains irregular and is still asymmetric also at the exit.

#### 3.8.2 Conductance of the lab-scale freeze-dryer. Influence of the shape of the entrance

**Fig. 24:**
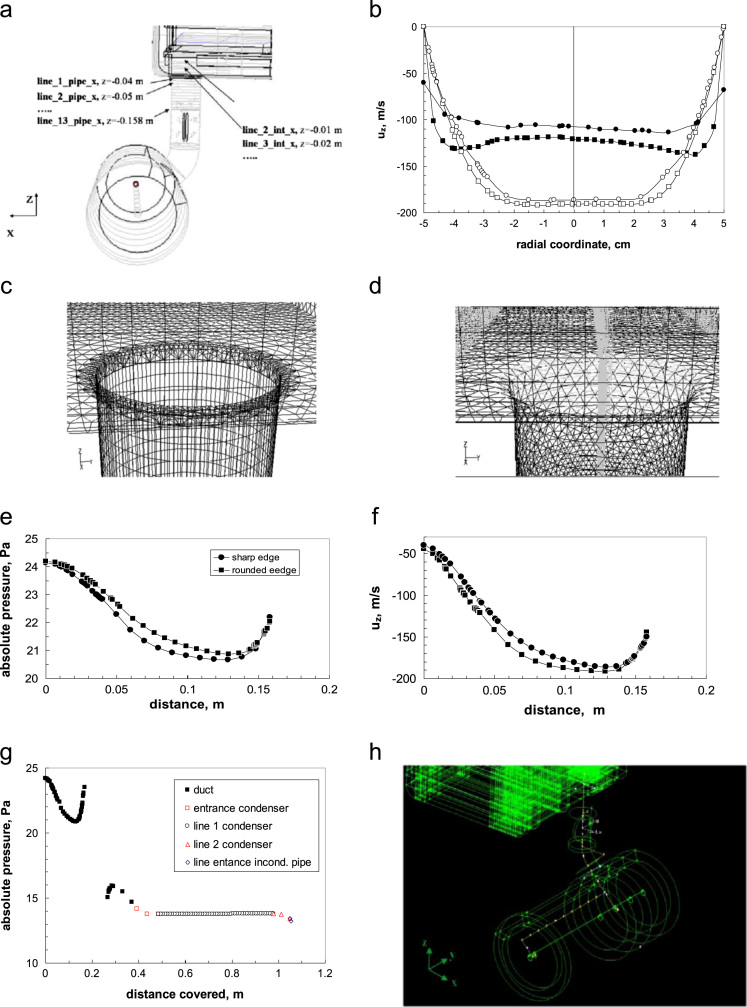
(a) Detail of the duct, with position of the profiles shown in Fig. 6 (bottom graphs) in Ref. [2]. (b) Radial velocity profiles at duct inlet (filled symbols) and at *z*=− 0.13 m (open symbols) in case of sharp entrance (square symbols) and rounded edge (circle symbols). (c–d) Details of the mesh geometry for the sharp entrance (left) and the rounded entrance (right). (e) Profile of the absolute pressure (Pa) along the axial direction of the duct. (f) Profile of the axial component of the fluid velocity, *v*_*z*_ (m/s) along the axial direction of the duct. (g) Absolute pressure along duct and condenser for the case with rounded entrance; the path considered is represented by the yellow line in (h).

The whole apparatus, including the condenser chamber, has been modelled in this case. Fig. 24a shows the details of the duct, with location of the plane where the radial pressure profiles shown in Fig. 6 (bottom graphs) of the article [2] are taken.

Two different entrances have been compared: a straight entrance and a rounded one. Two simulations have been carried out to investigate this effect, differing only in this geometric feature.

The details of the meshed geometries are shown in Fig. 24c and d, for the two cases.

The effect of the entrance type on the values of pressure and axial velocity along the duct axis is shown in Fig. 24e and f; the first straight tract of the duct, from the chamber outlet up to the disk valve (*z*=−0.158 m) is shown. The pressure drop in higher in the case of a sharp entrance: about 0.5 Pa more than in the case of the rounded entrance. The behavior is similar for the axial velocity (negative values are shown because the direction is from top to bottom). The increase of pressure at the end of the section considered corresponds to the reduction of velocity, caused by the presence of the obstacle (the open disk of the valve).

It must be evidenced that the profile drawn is just that one on the axis; the pressure at the wall keep decreasing, while the radial profile changes continuously (see Fig. 6 in Ref. [2]). There the radial profiles at different distances are shown: four in the chamber clearance, and 13 in the duct, each one distanced of 10 mm (for sake of clarity profiles corresponding to line 6, 8 and 9 have been omitted).

The influence of the shape of the entrance is shown in Fig. 24b: in case of sharp edges the entrance profile presents two lateral maxima, similarly to what observed in the larger apparatus, even if less marked. The rounded edge generates a flatter profile; the maximum velocity is lower, but it must be taken into account that the inlet area is slightly larger just at the inlet in this case.

The profile at the end of this section (at *z*=−0.13 m, with the origin of the axis in the chamber below the bottom shelf), just before the valve, is also shown; the velocity profile is still developing and some differences between the two cases are still evident.

The profile in the second part of the duct (and then in the condenser) can be seen in Fig. 24g; the gap in the graph is due to the presence of the butterfly valve. The complex behavior is again related to the developing pressure and velocity profile after the valve, with the change of section; the influence of the shape of the duct entrance is no longer significant.

#### 3.8.3 Fluid dynamics in the condenser

In Fig. 24g the profile of the absolute pressure along the whole path, is also shown, from the duct inlet to the condenser exit (as the center of the condenser is occupied by the exit pipe of the inert gases, the pressure is taken in the middle of the annular zone, as shown in Fig. 24h); the distance covered along the path is reported on the abscissa axis.

Different symbols and colors are used to represent the profiles along the different tracts of the path: the duct, the radial tract after the condenser entrance, the tract along the condenser (line 1 condenser), parallel to the condenser axis, the second radial tract (line 2 condenser) and the line in front of the incondensable gas pipe entrance (line entrance incond. pipe). This confirms that in this case there are no significant pressure gradients in the condenser, except just at the outlet.

**Fig. 25:**
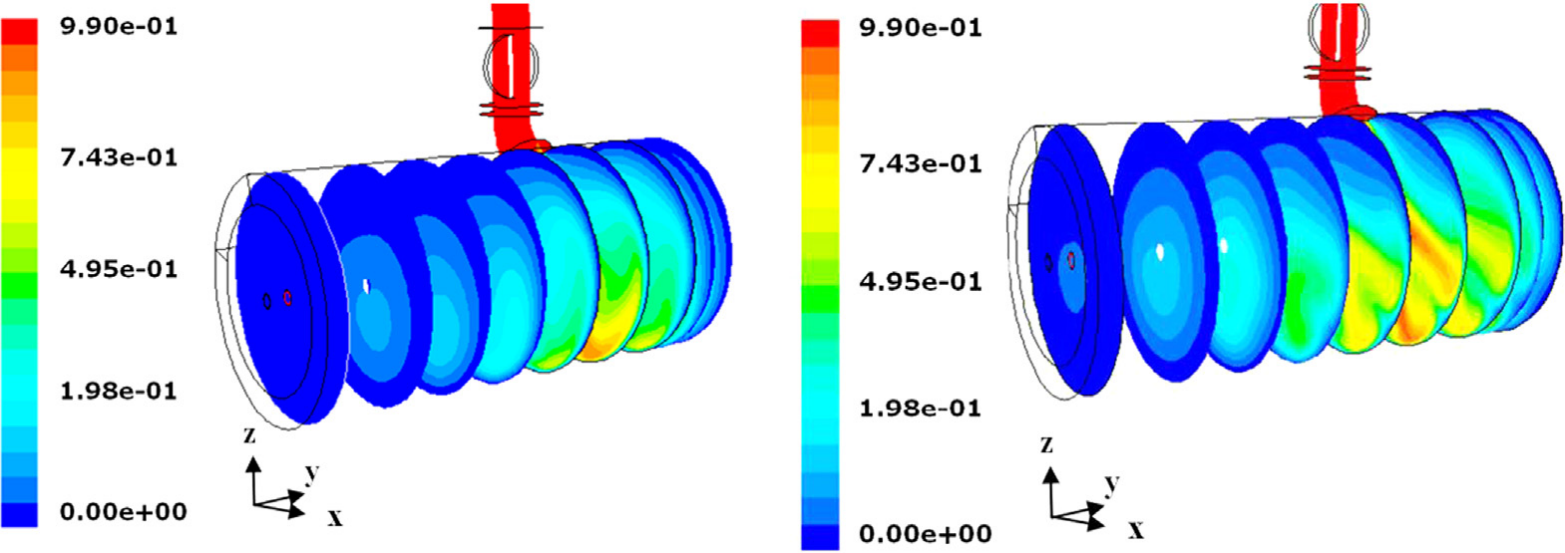
Zoom of the contours of the water mass fraction in the condenser, for the case with 5% inert gas (left) and 1% inert gas (right). Sublimation rate=1 kg h^−1^ m^−2^.

What changes along the condenser is the distribution of the water mass fraction, due to the icing at the wall surface. This can be clearly seen in Fig. 25, where two cases, with a different inert gas fraction, are compared; as mentioned in Refs. [1], [6], the inert gas is responsible for a blanketing effect in the condenser.

**Fig. 26:**
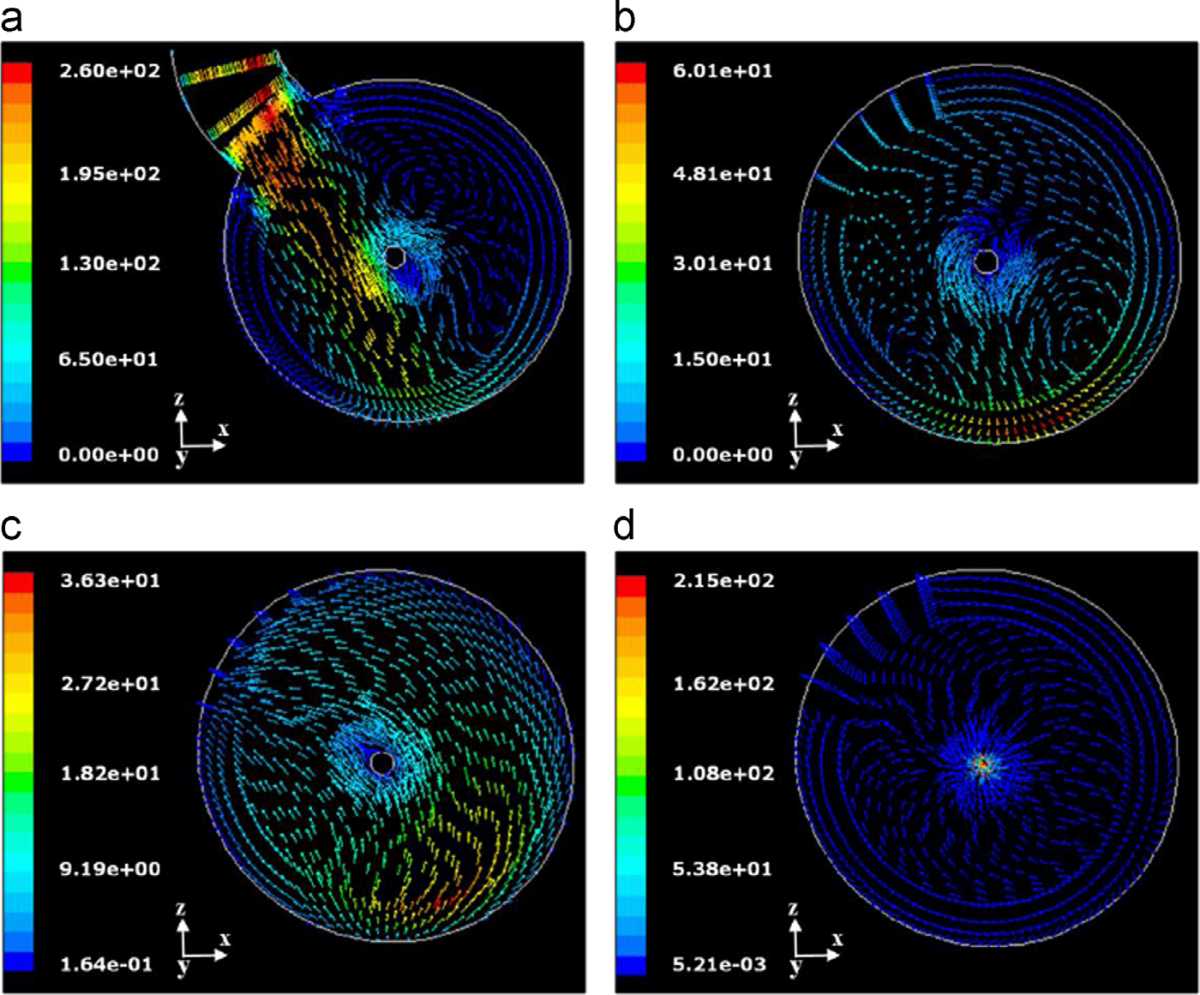
Velocity vectors on different condenser sections, taken from the bottom to the front: (a) at the entrance; (b) at 9 cm from the entrance, towards the front; (c) at 13 cm from the entrance, towards the bottom; (d) at incondensable gas pipe entrance. Sublimation rate=0.4 kg h^−1^ m^−2^, with 5% w/w inert gas.

CFD is also useful in this case to evidence the flow pattern in the apparatus. Fig. 26 shows, as an example, the velocity vectors of the gas mixture on different condenser sections.

### 3.9 Mushroom valve

Fluid dynamics data for different values of opening valve distance and mass flow rate, depending on the sublimation rate on the chamber shelves, are shown; finally a graphic correlation for the critical water flux as a function of the condenser pressure will be shown.

**Fig. 27:**
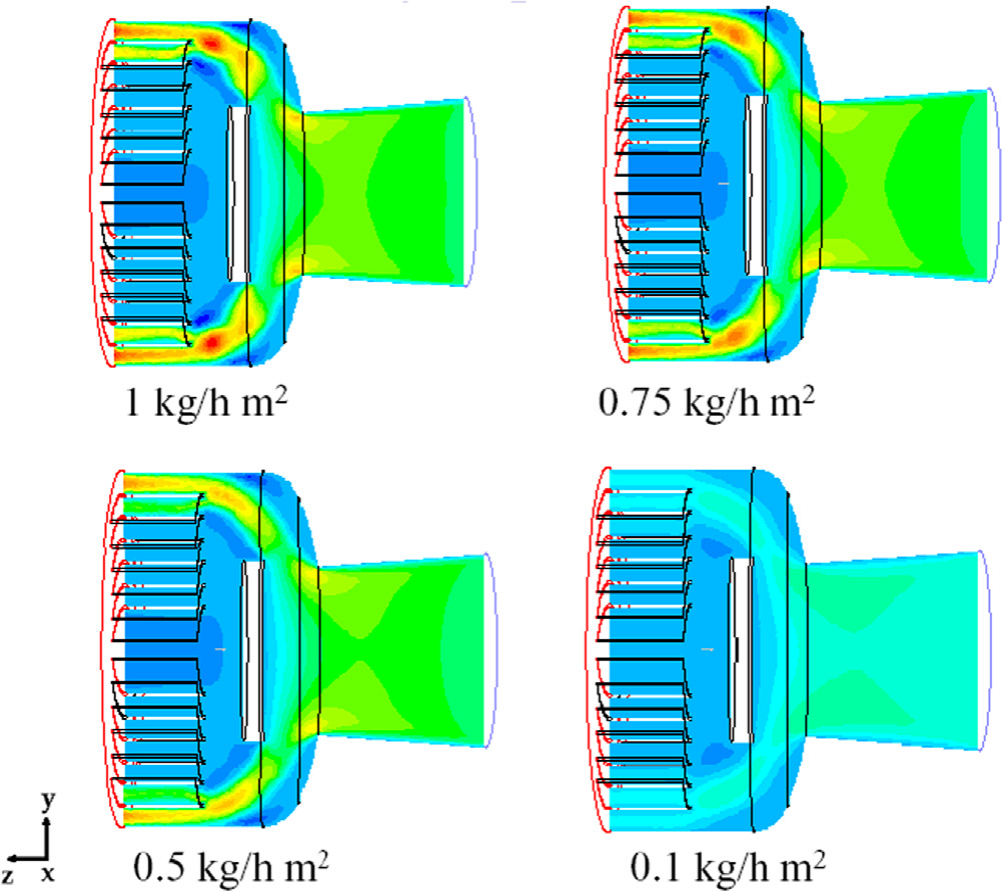
Mushroom valve (*l*_valve_=215 mm): axial velocity on plane *x*=0 for different values of the sublimating rate (17 sublimating shelves of the industrial scale freeze-dryer): green=180 m/s; yellow=250 m/s; red=480 m/s.

Fig. 27 shows the contour plots of the velocity along the plane *x*=0 for different values of sublimation rate: the values observed are proportional to the mass flow rate, but the qualitative behavior is similar; in all cases higher velocities (and corresponding higher mass flows) occur in the plane *y*=0 in the outer region.

**Fig. 28:**
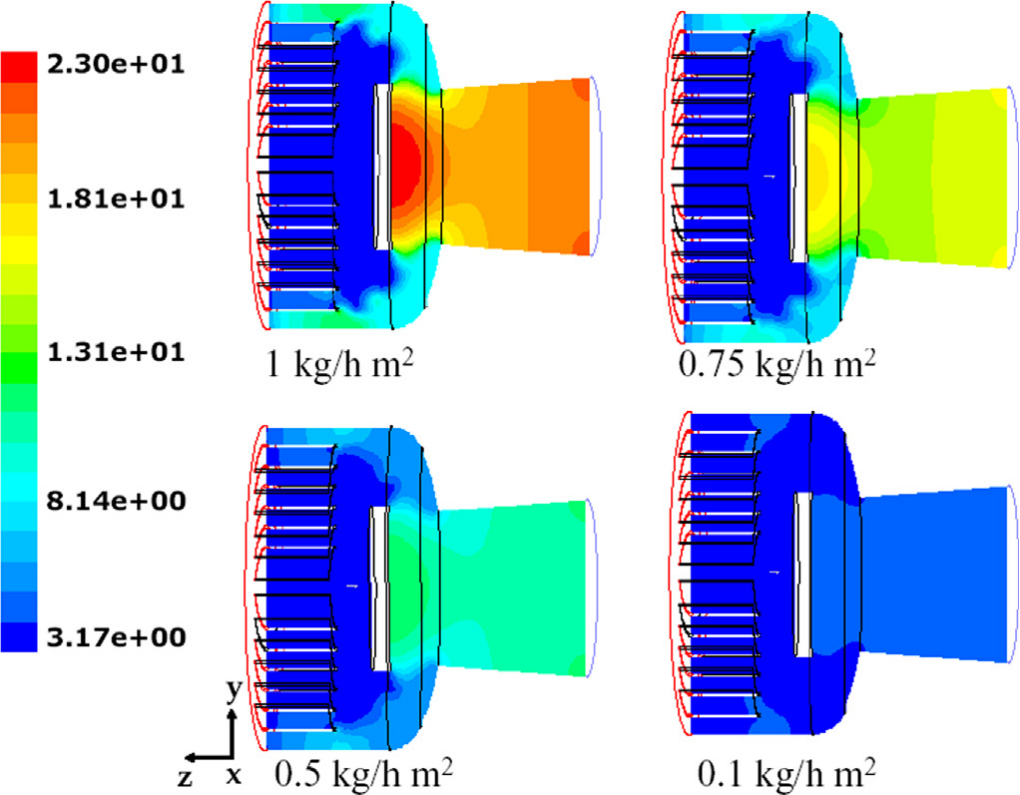
Mushroom valve (*l*_valve_=215 mm): absolute pressure on plane *x*=0 for different values of the sublimating rate (17 sublimating shelves of the industrial scale freeze-dryer).

Fig. 28 shows for one of the valve distances the pressure distribution in the system, for different values of the sublimation rate. The strong reduction in velocity caused by the presence of the disk determines a significant increase in the absolute pressure that locally, in correspondence of the disk, can be higher than the inlet value.

**Fig. 29:**
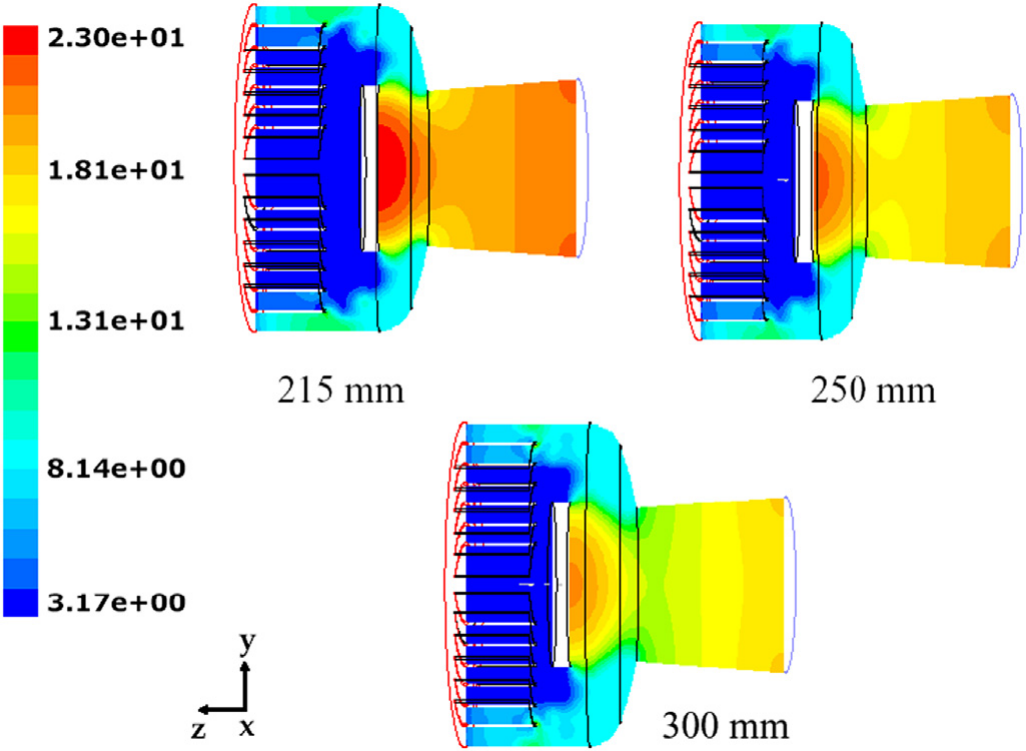
Mushroom valve: absolute pressure on plane *x*=0 for different values of the valve distance; 17 shelves of the industrial scale freeze-dryer, sublimating rate=1 kg h^−1^ m^−2^.

Fig. 29 shows the contour plots of the absolute pressure for the three configurations investigated, evidencing that the valve distance significantly affects the pressure drop, and in particular the pressure in the inlet zone: absolute pressure increases significantly in front of the valve disk, and the shorter the valve distance (that is the smaller the clearance for the passage of the vapor) the higher the pressure at the inlet; differences in the rear zone are much weaker.

To get a deeper insight into the hydrodynamics of the apparatus, and to better understand the influence of the vapor mass flow rate, the profiles of the variables of interest are shown.

**Fig. 30:**
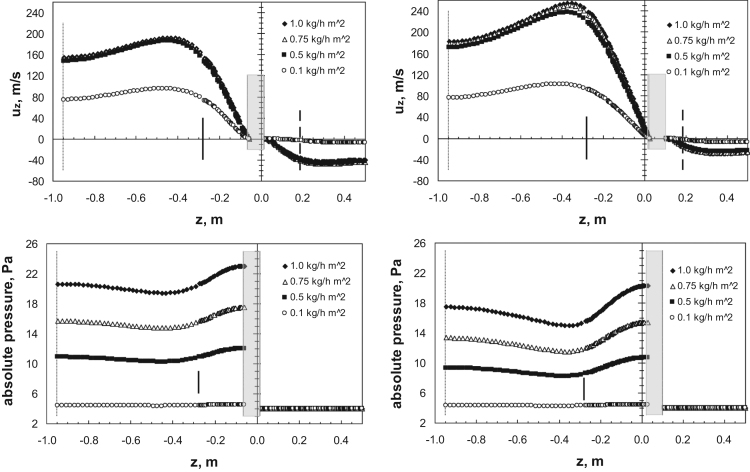
Upper graphs: axial profile along the central axis of the axial velocity component, for different sublimation rates. Bottom graphs: axial profile along the central axis of the absolute pressure, for different sublimation rates. Left graphs, *l*_valve_=215 mm; right graphs, *l*_valve_=300 mm. The position of the duct inlet (dash-dotted line), of the restricted section at vessel inlet (continuous bar), of the valve disk (grey area) and of the tube bundle front (dashed line) is shown for convenience. Sublimation rate=1 kg h^−1^ m^−2^ from 17 shelves of the industrial scale freeze-dryer.

Fig. 30 (upper graphs) compares the axial velocity of the vapor along the axis, from the inlet of the conic duct up to the first tube bundle section, for the two extreme valve-position configurations. It is evident that while at the lowest sublimation rate, when the flow is subcritical, the velocity is significantly lower than in the other cases, the three curves corresponding to the higher sublimation rates are almost overlapping. This apparently strange behavior can be easily explained, considering the results already shown in Fig. 28, from which it can be seen that the pressure in the inlet duct increases with mass flow rate; as the inlet pressure increases practically in a linear way, then the increase in mass flow rate is compensated by the increase in the density value, leading approximately to the same velocity value. This is true not only on the axis, but in all the zone upstream the disk, while around and beyond the disk the flow field velocity is dependent on the mass flow rate (as shown in Fig. 27).

Fig. 30 (bottom graphs) compares the absolute pressure axial profiles for the two configurations; it is evident that while behind the valve zone the pressure remains constant and practically equal to the exit value, for all flow rates, in the inlet it increases significantly with the mass flow rate. The increase is higher for the valve with a lower disk distance, because this causes a stronger flow restriction, but the variation in the zone before the disk (that is similar to that observed for the axial velocity) is larger in the valve with larger clearance.

**Fig. 31:**
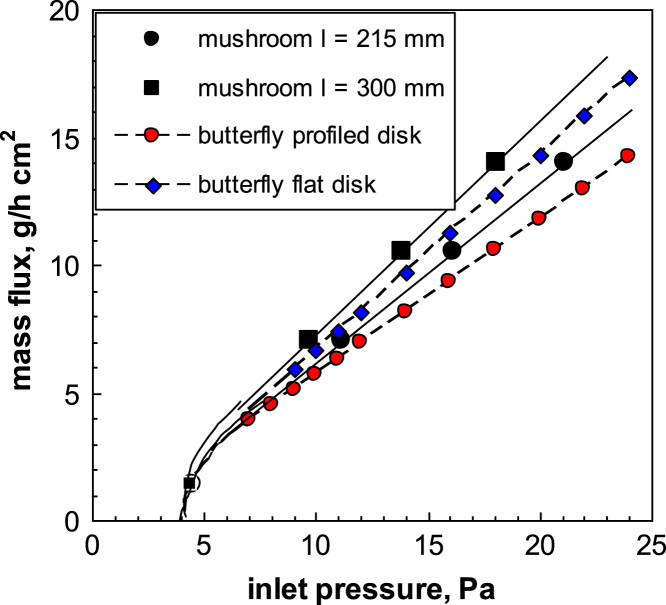
Comparison of mushroom and butterfly valve conductance; *P_out_*=4 Pa.

It is interesting to compare the behavior of the mushroom valve with that of the butterfly valves investigated before. Fig. 31 shows that the conductance of mushroom and butterfly valves is comparable. It appears in particular that the conductance of the simple butterfly valve with flat disk in intermediate between that of the mushroom valve with smallest and largest clearance, while that of the butterfly valve with profiled disk is smaller.
