# Supplementary material for: Computational Fluid Dynamics data for improving freeze-dryers design
Source: Data Brief. 2018 May 31;19:1181–213. doi: 10.1016/j.dib.2018.05.141 (PMC6140831; doi:10.1016/j.dib.2018.05.141)
Supplement: Supplementary file 1 — Supplementary material [file mmc1.docx]

**Conflict Of Interest**

The authors declare that there is no conflict of interest.
